# Public responses to the Salisbury Novichok incident: a cross-sectional survey of anxiety, anger, uncertainty, perceived risk and avoidance behaviour in the local community

**DOI:** 10.1136/bmjopen-2019-036071

**Published:** 2020-09-25

**Authors:** G James Rubin, Rebecca Webster, Richard Amlot, Holly Carter, Dale Weston, Simon Wessely

**Affiliations:** 1 Institute of Psychiatry, Psychology and Neuroscience, King's College London, London, UK; 2 Department of Psychology, The University of Sheffield, Sheffield, UK; 3 Emergency Response Department, Public Health England Porton, Salisbury, Wiltshire, UK

**Keywords:** public health, risk management, toxicology

## Abstract

**Objectives:**

Malicious incidents involving chemical agents sometimes trigger high public concern. We aimed to (1) identify levels of emotion, perceived risk and behaviour change with regard to visiting Salisbury, 1 month after three people were poisoned with a nerve agent; and (2) test whether factors including receipt of information, beliefs about personal exposure and trust in government were associated with these outcomes.

**Design:**

A cross-sectional telephone survey of a random sample of Salisbury residents.

**Setting:**

Conducted between 5 and 13 April 2018.

**Participants:**

500 residents aged 18 or over.

**Outcome measures:**

Self-reported anxiety, anger, uncertainty, perceived risk to self and avoidance of Salisbury.

**Results:**

Any degree of anxiety, anger and uncertainty was reported by 40.6%, 29.8% and 30.6% of participants, respectively. For the majority, the level of emotion reported was mild. Only 7.0% met the criteria for high anxiety and 5.2% reported feeling any risk to their health, whereas 18.6% reported avoiding Salisbury. Factors associated with avoidance of Salisbury included being female, unable to rule out exposure for oneself or of loved ones, believing the incident was targeted against the general public, and lower trust in the government and responding agencies. Hearing a lot or a little about the recovery support (eg, financial packages), as opposed to nothing at all, and being satisfied with this information were associated with reduced avoidance.

**Conclusions:**

Although the March 2018 Salisbury incident had a relatively modest impact on emotion and risk perception in the community, the number who reported avoiding the city was notable. In this, and in future incidents, assuring people that contamination resulted from a targeted, rather than indiscriminate, incident; demonstrating that contamination is contained within specific areas; improving communication about any financial support; and promoting trust in responding agencies should help provide additional reassurance to the community.

**Strengths and limitations of this study**

- The study sample was demographically representative of adult residents living in Salisbury, UK in terms of age and gender.
- The study took place shortly after the Salisbury Novichok incident, and data collection took place over a short period of time.
- While it is possible that the sample was biased towards people with a specific interest in the incident, we attempted to reduce this by not revealing the full topic of the survey until after initial consent had been obtained.
- This study replicated previous findings about the importance of the perceived extensiveness of contamination and the perceived motivation for an incident in determining emotional and behavioural responses and risk perception.
- The response rate was extremely low, and although quota sampling may have mitigated the effects of this, whether the surveys are psychologically representative of the general population is unclear.

## Introduction

Public health incidents involving chemical, biological, radiological or nuclear (CBRN) agents can sometimes trigger seemingly disproportionate levels of distress among the affected community. Previous incidents have witnessed high levels of anxiety or anger,^1^ spontaneous evacuations or attempts to avoid potentially contaminated areas,^2 3^ stigmatisation of people seen as contaminated,^4 5^ high levels of help-seeking among people who have not been exposed to the agent,^6^ and economic and social upheavals.^7^

Attempts by responding agencies to reduce these effects often focus on improving communication with an affected community by identifying and addressing specific concerns (eg, refs 8 9) and by building trust with those affected (eg, refs 10 11). Research in this area has resulted in generic guidelines that can be used to assist in the acute phase of these responses, for example by identifying topics that members of the public are likely to have particular misgivings about, such as perceived exposure to a toxic substance.^12 13^ Less research has focused on the importance of other factors that may become relevant once the acute phase of a CBRN incident has passed, for example the importance of communication about the clean-up operation or the level of public satisfaction with any financial support that is subsequently made available.

The literature on CBRN incidents is also problematic because it often relies on the use of hypothetical scenarios, rather than the study of actual incidents. For example, in a 2012 systematic review of communication research relating to the deliberate use of CBRN material,^12^ out of the 33 studies identified only 12 investigated genuine incidents, 11 of which related to the US anthrax attacks of 2001. The 12th explored the reactions of the London population to the use of radioactive polonium 210 in the apparent assassination of former Russian intelligence officer Alexander Litvinenko, an incident which resulted in a major public health response after a restaurant and hotel bar were found to be contaminated.^9^ The study found that increased concern among members of the public was linked to the perceived motivation underlying the incident (ie, that it had been targeted at the wider public rather than at a single individual) and to a belief that contamination might occur away from the areas that had been cordoned off by the emergency services.

While studies on genuine incidents involving the use of CBRN agents are rare, rarer still are studies that are able to reproduce previous findings. The idiosyncrasies of each incident in terms of the population affected, public familiarity with the agent and the media narrative surrounding the incident often make it difficult to compare results. But on 4 March 2018, an incident occurred in England which bore remarkable similarities to the polonium 210 incident. Sergei Skripal, an ex-Russian intelligence officer, his daughter and a police officer became seriously ill following exposure to the nerve agent Novichok in what the UK Government described as an attempted assassination. The incident occurred in the small English city of Salisbury (population: 40 000), whose residents suddenly found themselves at the centre of a major police investigation, media furore and diplomatic crisis. Several areas of the city were cordoned off by police both to preserve forensic evidence and to prevent other members of the public from being exposed to the agent. Investigations initially focused on a pub and restaurant that were visited by the Skripals shortly before they became ill.

A week after the cordons went up, the Chief Medical Officer for England advised that, as a precautionary measure, members of the public who had been in the pub or restaurant at the time of the incident should wash the clothes they had been wearing, wipe or wash personal items such as mobile phones or jewellery, and seal dry-clean-only clothing in plastic bags. It was later announced that dry-clean-only clothing would be collected and incinerated. As the clean-up operation continued, a package of financial measures were announced by the government to support individuals and businesses that had been affected by a decline in visitors into the city. It was not until 1 March 2019 that the decontamination efforts in Salisbury finally came to an end.

In this study, we used a telephone survey of the local population of Salisbury conducted 1 month after the incident began in order to (1) identify levels of anxiety, anger, uncertainty, perceived risk and changes in behaviour regarding visiting Salisbury; (2) test whether factors that may be particularly relevant in the acute stage of the incident (amount heard about the incident, perceived exposure of self, perceived exposure of significant others, perceived motivation for the incident) were associated with these outcomes; and (3) test whether factors related to the recovery stage of the incident were associated with these outcomes, specifically the amount heard about the clean-up or recovery effort, the satisfaction with this information or the level of support offered, the expected length of the clean-up, the level of impact on the participant’s daily lives, or the level of trust in the government and responding agencies.

## Methods

### Design

Between 5 and 13 April 2018, ICM Unlimited conducted a telephone survey of 500 residents living in Salisbury, UK. This sample size provided a total 95% CI of approximately ±4% for each prevalence estimate. To achieve a broadly representative sample, ICM Unlimited used a random approach to ensure that all landline telephone numbers within the geographical region had an equal chance of being called, while quotas were set for the sample based on age and gender based on 2011 census data. To reduce selection bias, participants were initially informed that the survey related to ‘issues affecting the UK’ and were only informed after providing consent that the particular issues of interest were the recent events in Salisbury. Interviews typically lasted around 10 min. Calls were made during the working day, during the evenings and on weekends to reduce selection bias associated with working outside the home.

### Patient and public involvement

No patients were involved.

### Participants

In order to take part, respondents were required to be 18 years or older, to live in Salisbury and surrounding areas (postcode areas SP1, SP2, SP3, SP4, SP5 and SP6), and to speak English.

### Outcomes

The online supplemental material provides a copy of the wording used for all items in the survey.

10.1136/bmjopen-2019-036071.supp1 Supplementary data

Anxiety was measured using an adapted version of the Six-Item State Trait Anxiety Inventory (STAI-6),^14^ which asked participants to report how they had been feeling over the past week ‘when thinking about the incident in Salisbury’. The STAI-6 provides scores from 6 (least anxiety) to 24 (most anxiety). In line with previous work on public responses to major incidents,^1 15^ we categorised people who scored 12 or more as having anxiety about the incident and those who scored 18 or more as having high anxiety. Two additional items were added to the end of the inventory to assess anger and uncertainty. These were analysed separately, with responses of ‘moderately’ or ‘very much’ being taken as indicating the presence of anger or uncertainty.

Perceived risk was measured using a single item adapted from a previous work on the polonium 210 incident^9^ asking participants ‘to what degree do you feel your health is at risk as a consequence of the recent incident in Salisbury’. Responses were given on a scale of 0 (not at all) to 4 (a lot). As with the polonium 210 incident, we categorised people who gave a response of 3 or 4 as perceiving a personal health risk.

Changes in behaviour regarding visiting Salisbury were assessed using the item ‘Since the incident in Salisbury, have you deliberately reduced the amount you go into Salisbury?’ Participants could respond ‘yes’ or ‘no’.

### Predictor measures related to the acute stage of the incident

Participants were asked how much they had seen, heard or read about the incident, with response options being a lot, a little and nothing at all.

In order to assess perceived exposure, participants were asked whether they believed that they, or a close family member or someone dear to them, had been exposed to ‘any of the chemical that was used in this incident’, with responses definitely yes, probably yes, not sure, probably no and definitely no.

In order to assess perceptions about the motives underlying the incident, we adapted a single item from the polonium 210 study^9^ which asked participants whether they thought the incident was intended to harm only a single person, a small number of people or the wider public.

### Predictor measures related to the recovery stage

Participants were asked how much they had seen, heard or read about the clean-up process in Salisbury and about recovery support, ‘for example the financial support package the government has made available’. For both questions, possible responses were a lot, a little and nothing at all. For those aware of the recovery support package, follow-up questions asked the extent to which they felt the information they had received was clear, timely, sufficient and useful. These items were summed to give a scale with acceptable internal reliability (Cronbach’s alpha 0.78), from 4 (least satisfied) to 20 (most satisfied). Participants were also asked their level of agreement with statements that sufficient support was being offered by the government to individuals directly affected, businesses directly affected, local residents across Salisbury and local business across Salisbury. Again, these items were summed to give a scale with acceptable internal reliability (Cronbach’s alpha 0.88), from 4 (least satisfied) to 20 (most satisfied).

Participants were asked a single open-ended item on their expectations for the length of the clean-up process.

Impact on the participant’s financial or daily life was assessed by asking whether ‘your ability to go to work has been affected’, ‘you own a business that has been affected’, ‘your income has been affected’, or ‘there has been some other, serious, disruption to your day to day life’.

Trust in the official response was assessed using six items developed for use in an infectious disease outbreak,^16^ which assessed whether, in relation to the incident, the participant thought the government and official agencies were doing a good job, had enough resources, had the necessary knowledge, were acting in the public’s best interests, were managing the incident in a fair way, and whether they felt confident about their ability to deal with the incident. Responses to these items showed acceptable reliability (Cronbach’s alpha 0.74) and were summed to provide a score of 6 (least trust) to 18 (most trust), and then dichotomised into scores of low (12 or below) versus high (13 or up) trust.

### Statistical analysis

For all analyses we coded responses of ‘don’t know’ or ‘not applicable’ as missing data.

We used logistic regressions to calculate adjusted ORs to test the association between gender and age (controlled for each other) and each of the outcome variables, as well as for each predictor measure (controlling for gender and age) and each outcome variable.

For perceived exposure variables, we compared participants who believed they had probably or definitely been exposed or who were not sure, against those who believed they had probably or definitely not been exposed. For perceived impact, we compared those who reported impact on any aspect of their life with those who reported no impact.

Although topline data in the online supplemental materials are reported weighted, for ease of interpretation, all analyses were conducted using unweighted data.

## Results

### Response rates

In order to obtain 500 valid responses, ICM Unlimited made a total of 18 753 calls, resulting in contact with 6808 people who were within quota (7.3%). Low response rates are typical of this style of telephone survey and are not necessarily indicative of high response bias.^17^ Two hundred and eighty (56.0%) participants were female and 220 (44.0%) were male. This was roughly in line with 2011 census data for Salisbury (52% female, 48% male).^18^ One hundred and sixty-eight (33.6%) participants were aged 18–44, 195 (39.0%) were aged 45–64, and 137 (27.4%) were 65 or over. This showed an under-representation of individuals aged 18–44 years old compared with census data (43%). Seventeen (3%) participants reported having been in the pub or restaurant at the centre of incident.

### Main outcomes

The main outcomes are summarised in table 1. Two hundred and three (40.6%) participants were defined as having some level of anxiety, while anger and uncertainty were reported by 149 (29.8%) and 153 (30.6%) participants, respectively. Only 35 (7.0%) participants met the criteria for high anxiety and only 26 (5.2%) reported feeling that there was any risk to their own health. Ninety-two (18.6%) reported having deliberately reduced the amount they go into Salisbury.

**Table 1:** Emotional and behavioural reactions to the Salisbury poisoning

Emotional or behavioural outcome	Reporting the outcome (n)/valid response, n (%)
High or moderate anxiety	203/500 (40.6)
High anxiety	35/500 (7.0)
Anger	149/500 (29.8)
Uncertainty	153/500 (30.6)
Perceived personal risk	26/498 (5.2)
Reduced amount in going to Salisbury	92/494 (18.6)

### Acute predictors of outcomes

Table 2 shows the associations between the various acute stage predictor variables and emotional response. In general, older adults were more likely to report anxiety and anger, while women were more likely to report anxiety. People who were unable to rule out the possibility that they themselves had been exposed to the chemical agent were more likely to report anxiety, anger and uncertainty, while people who were unable to rule out exposure among friends or relatives were more likely to report anxiety and uncertainty. Those who believed the incident was targeted at the wider public were more likely to report uncertainty than people who felt it was targeted at a single individual.

**Table 2:** Association between acute stage predictor measures and emotional outcomes

Variable	Variable levels	Participants, n (%)	Reporting anxiety, n (%)	OR (95% CI)	Reporting anger, n (%)	OR (95% CI)	Reporting uncertainty, n (%)	OR (95% CI)
Age	65+	137 (27.4)	72 (52.6)	**2.2 (1.4 to 3.5)**	65 (40.9)	**2.6 (1.5 to 4.2)**	46 (33.6)	1.5 (0.9 to 2.5)
	45–64	195 (39.0)	72 (36.9)	1.1 (0.7 to 1.7)	57 (29.2)	1.5 (0.9 to 2.4)	64 (32.8)	1.4 (0.9 to 2.2)
	18–44	168 (33.6)	59 (35.1)	Reference	36 (21.4)	Reference	43 (25.6)	Reference
Gender	Female	280 (56.0)	129 (46.1)	**1.8 (1.3 to 2.6)**	84 (30.0)	1.1 (0.7 to 1.6)	89 (31.8)	1.2 (0.8 to 17)
	Male	220 (44.0)	74 (33.6)	Reference	65 (29.5)	Reference	64 (29.1)	Reference
How much heard	A lot	449 (90.2)	180 (40.1)	0.8 (0.5 to 1.5)	129 (28.7)	0.7 (0.3 to 1.2)	140 (31.2)	1.6 (0.8 to 3.2)
	A little/nothing	49 (9.8)	23 (46.9)	Reference	19 (38.8)	Reference	11 (22.4)	Reference
Belief in exposure (self)	Definitely, probably or not sure	21 (4.2)	16 (76.2)	**5.5 (2.0 to 15.7)**	11 (52.4)	**2.9 (1.2 to 7.0)**	14 (66.7)	**4.9 (1.9 to 12.4)**
	Definitely or probably not	479 (95.8)	187 (39.0)	Reference	138 (28.8)	Reference	139 (29.0)	Reference
Belief in exposure (others)	Definitely, probably or not sure	44 (8.8)	27 (61.4)	**2.6 (1.3 to 4.9)**	18 (40.9)	1.8 (0.96 to 3.5)	26 (59.1)	**3.8 (2.0 to 7.2)**
	Definitely or probably not	456 (91.2)	176 (38.6)	Reference	131 (28.7)	Reference	127 (27.9)	Reference
Incident intended to harm…	Wider public	31 (6.2)	18 (58.1)	2.1 (0.96 to 4.5)	13 (41.9)	1.5 (0.7 to 3.2)	16 (51.6)	**2.5 (1.2 to 5.3)**
	Small number of specific people	187 (37.4)	80 (42.8)	1.2 (0.8 to 1.8)	53 (28.3)	0.9 (0.6 to 1.4)	56 (29.9)	1.0 (0.7 to 1.5)
	A single person	265 (54.9)	101 (38.1)	Reference	81 (30.6)	Reference	78 (29.4)	Reference

ORs are adjusted for age and gender.

Bold alues significant at p<.05

Although the small number of people categorised as having high anxiety prevented us from conducting detailed statistical analyses, it was notable that most people in this group had not been to a contaminated location and reported believing that neither they nor any close friends or relatives had been exposed to the nerve agent.

Table 3 shows the association between the acute stage predictors and perceived risk to self or behaviour change. People who could not rule out that they themselves had been exposed to the chemical agent were more likely to feel that their health was at risk and to have avoided visiting Salisbury. The same pattern of findings was also true for those who could not rule out exposure of friends or family members, and those who felt the incident was targeted at the wider public rather than at a single person. Being female was only associated with avoidance of Salisbury, whereas participants who had heard little or no information about the incident were more likely to feel that their health was at risk.

**Table 3:** Association between acute stage predictor measures, risk perception and behaviour

Variable	Variable levels	Participants, n (%)	Reporting personal risk perception, n (%)	OR (95% CI)	Reporting avoiding Salisbury, n (%)	OR (95% CI)
Age	65+	137 (27.4)	9 (6.6)	1.9 (0.7 to 5.6)	20 (15.2)	0.7 (0.4 to 1.2)
	45–64	195 (39.0)	11 (5.7)	1.6 (0.6 to 4.4)	35 (17.9)	0.8 (0.5 to 1.3)
	18–44	168 (33.6)	6 (3.6)	Reference	37 (22.2)	Reference
Gender	Female	280 (56.0)	16 (5.7)	1.3 (0.6 to 3.0)	61 (22.1)	**1.7 (1.1 to 2.7)**
	Male	220 (44.0)	10 (4.6)	Reference	31 (14.2)	Reference
How much heard	A lot	449 (90.2)	20 (4.5)	**0.3 (0.1 to 0.9)**	84 (18.8)	1.2 (0.5 to 2.7)
	A little/nothing	49 (9.8)	6 (12.2)	Reference	8 (17.4)	Reference
Belief in exposure (self)	Definitely, probably or not sure	21 (4.2)	6 (28.6)	**9.3 (3.2 to 26.9)**	9 (42.9)	**3.5 (1.4 to 8.8)**
	Definitely or probably not	479 (95.8)	20 (4.2)	Reference	83 (17.5)	Reference
Belief in exposure (others)	Definitely, probably or not sure	44 (8.8)	7 (15.9)	**4.4 (1.7 to 11.4)**	19 (45.2)	**4.0 (2.0 to 7.7)**
	Definitely or probably not	456 (91.2)	19 (4.2)	Reference	73 (16.2)	Reference
Incident intended to harm…	Wider public	31 (6.2)	7 (22.6)	**7.8 (2.6 to 22.9)**	13 (44.8)	**5.0 (2.2 to 11.3)**
	Small number of specific people	187 (37.4)	8 (4.3)	1.2 (0.5 to 3.4)	37 (20.2)	1.4 (0.9 to 2.3)
	A single person	265 (54.9)	9 (3.4)	Reference	40 (15.1)	Reference

ORs are adjusted for age and gender.

**Bold** = significant at p<.05

### Recovery stage predictors of outcomes

Tables 4 and 5 show the associations between recovery stage predictors and the outcomes. Greater satisfaction with the information given out about the recovery support was associated with lower levels of anxiety and uncertainty, while participants who reported having heard nothing about the recovery support or who were less satisfied with the support available had a greater likelihood of avoiding Salisbury. Those who thought the clean-up would take less than 1 month were more likely to perceive a lower risk to self. Lower trust in the government and official agencies was associated with a greater likelihood of anxiety, uncertainty, perceived risk to self and avoiding Salisbury.

**Table 4:** Association between recovery predictor measures and emotional outcomes

Variable	Variable levels	Participants, n (%)	Reporting anxiety, n (%)	OR (95% CI)	Reporting anger, n (%)	OR (95% CI)	Reporting uncertainty, n (%)	OR (95% CI)
How much heard—clean-up	A lot	169 (34.3)	75 (44.4)	0.9 (0.5 to 1.5)	56 (33.1)	1.3 (0.7 to 2.5)	56 (33.1)	1.3 (0.7 to 2.6)
	A little	257 (52.2)	95 (37.0)	0.7 (0.4 to 1.2)	73 (28.4)	1.2 (0.6 to 2.2)	78 (30.4)	1.2 (0.7 to 2.3)
	Nothing	66 (13.4)	30 (45.5)	Reference	17 (25.8)	Reference	17 (25.8)	Reference
How much heard—recovery	A lot	96 (20.0)	35 (36.5)	**0.5 (0.3 to 0.9)**	30 (31.3)	0.9 (0.5 to 1.5)	25 (26.0)	0.6 (0.3 to 1.04)
	A little	235 (49.0)	93 (39.6)	0.7 (0.4 to 1.03)	70 (29.8)	0.9 (0.5 to 1.4)	72 (30.6)	0.8 (0.5 to 1.2)
	Nothing	149 (31.0)	69 (46.3)	Reference	45 (30.2)	Reference	52 (34.9)	Reference
Satisfaction with recovery information	Scale of 4 (least) to 20 (most)	Mean score: 13.2 (SD 3.8)	Not applicable	1.0 (0.9 to 1.04)	Not applicable	0.9 (0.9 to 1.002)	Not applicable	1.0 (0.9 to 1.07)
Satisfaction with support offered	Scale of 4 (least) to 20 (most)	Mean score: 12.9 (SD 3.4)	Not applicable	**0.9 (0.9 to 0.99)**	Not applicable	1.0 (0.9 to 1.02)	Not applicable	**0.9 (0.9 to 0.98)**
Expected length of clean-up	Less than a month	108 (29.9)	39 (36.1)	0.8 (0.5 to 1.4)	28 (25.9)	0.9 (0.5 to 1.5)	40 (37.0)	1.6 (0.9 to 2.8)
	1–2 months	124 (34.3)	50 (40.3)	1.0 (0.6 to 1.6)	40 (32.3)	1.2 (0.7 to 2.1)	39 (31.5)	1.3 (0.8 to 2.2)
	2 months or more	129 (35.7)	52 (40.3)	Reference	37 (28.7)	Reference	34 (26.4)	Reference
Impact on daily life	Some impact	68 (13.6)	33 (48.5)	1.5 (0.9 to 2.6)	23 (33.8)	1.3 (0.7 to 2.2)	25 (36.8)	1.4 (0.8 to 2.4)
	No impact	432 (86.4)	170 (39.4)	Reference	126 (29.2)	Reference	128 (29.6)	Reference
Trust in government	Low trust	67 (13.4)	35 (52.2)	**1.8 (1.04 to 3.0)**	23 (34.3)	1.3 (0.8 to 2.3)	24 (35.8)	1.3 (0.8 to 2.3)
	High trust	433 (86.6)	168 (38.8)	Reference	126 (29.1)	Reference	129 (29.8)	Reference

ORs are adjusted for age and gender.

**Bold** = significant at p<.05

**Table 5:** Association between recovery predictor measures, risk perception and behaviour

Variable	Variable levels	Participants, n (%)	Reporting personal risk perception, n (%)	OR (95% CI)	Reporting avoiding Salisbury, n (%)	OR (95% CI)
How much heard—clean-up	A lot	169 (34.3)	9 (5.3)	1.1 (0.3 to 4.1)	32 (19.2)	0.8 (0.4 to 1.6)
	A little	257 (52.2)	13 (5.1)	1.1 (0.3 to 3.9)	45 (17.6)	0.7 (0.4 to 1.4)
	Nothing	66 (13.4)	3 (4.6)	Reference	15 (23.1)	Reference
How much heard—recovery	A lot	96 (20.0)	3 (3.1)	0.3 (0.1 to 1.1)	12 (12.5)	**0.4 (0.2 to 0.8)**
	A little	235 (49.0)	11 (4.7)	0.5 (0.2 to 1.1)	35 (15.2)	**0.5 (0.3 to 0.8)**
	Nothing	149 (31.0)	12 (8.2)	Reference	42 (28.6)	Reference
Satisfaction with recovery information	Scale of 4 (least) to 20 (most)	Mean score: 13.2 (SD 3.8)	Not applicable	0.9 (0.8 to 1.0)	Not applicable	1.0 (0.9 to 1.1)
Satisfaction with support offered	Scale of 4 (least) to 20 (most)	Mean score: 12.9 (SD 3.4)	Not applicable	1.0 (0.9 to 1.2)	Not applicable	**0.9 (0.8 to 0.96)**
Expected length of clean-up	Less than a month	108 (29.9)	1 (0.9)	**0.1 (0.01 to 0.7)**	15 (14.2)	0.6 (0.3 to 1.1)
	1–2 months	124 (34.3)	6 (4.8)	0.5 (0.2 to 1.3)	27 (21.8)	0.9 (0.5 to 1.7)
	2 months or more	129 (35.7)	12 (9.3)	Reference	29 (22.7)	Reference
Impact on daily life	Some impact	68 (13.6)	6 (8.8)	2.0 (0.8 to 5.3)	17 (25.8)	1.7 (0.9 to 3.1)
	No impact	432 (86.4)	20 (4.7)	Reference	75 (17.5)	Reference
Trust in government	Low trust	67 (13.4)	11 (16.7)	**5.7 (2.5 to 13.0)**	21 (31.8)	**2.3 (1.3 to 4.1)**
	High trust	433 (86.6)	15 (3.5)	Reference	71 (16.6)	Reference

ORs are adjusted for age and gender.

**Bold** = significant at p<.05

## Discussion

One month following the Novichok incident in Salisbury, many members of the local community reported some degree of anxiety (40.6%), anger (29.8%) or uncertainty (30.6%). By way of comparison, using the same scale, 23.7% of the British population reported some anxiety at the very earliest stages of the swine influenza outbreak.^15^ The number reporting high anxiety was relatively low, however, at 7.0%. Again, for comparison, the number reporting high anxiety among the general public of Great Britain during the early stages of the swine influenza outbreak was 2.1%,^15^ while among British nationals in Japan at the time of the Fukushima nuclear disaster it was 29.7%.^1^ Levels of perceived risk were also low (5.2%) and slightly lower than those found during the polonium 210 incident (11.7%).^9^ Despite emotional responses being relatively low however, behaviour change was apparent, with 18.6% of people from the local area reporting deliberately reducing the amount they went into Salisbury. This is similar to the 20% of Londoners who reported intending to going into central London less often after the 7/7 bombings.^19^

Our analyses suggested that there were three core issues underlying these findings. First, a pattern of associations was identified between the outcomes and believing that exposure might have occurred to oneself or a loved one, or feeling that the incident had been targeted at the wider public. Tellingly, most people reporting high anxiety had not been present within an area that was later cordoned off. Similar findings were also observed during the polonium 210 incident^9^ and suggest a level of concern that contamination may deliberately or accidentally occur beyond the areas identified by the responding agencies. Emphasising that this is unlikely should reduce levels of concern among the population, but responding agencies may need to exercise caution about this. In this incident, on 3 July 2018, two further members of the public became ill following accidental exposure to a source of Novichok from a location outside of the initial cordons. One subsequently died. As a result, multiple new locations were cordoned off by police, and members of the public were once again issued with advice about washing clothes and possessions.

The second core issue underlying concern related to the level of support being provided to the community during the recovery phase. The incident had implications for the economic well-being of the community, with local businesses and their employees affected by the presence of police cordons and a reduction in the number of visitors to the city. Although several measures were introduced to offset this, at the time of our survey most people had heard little or nothing about them. This in turn appeared to underlie some of the anxiety and uncertainty reported by residents. Interestingly, an association was also found between having heard more about the recovery package or satisfaction with the support available, and being less likely to avoid Salisbury. Whether the information provided reduced concerns about visiting the city or whether people who visited Salisbury were more likely to encounter information about the recovery effort is unclear. Nonetheless, ensuring that communication about recovery support is widely disseminated should be beneficial in future incidents.

The third core issue related to trust. Strong associations were observed between greater trust in the government and responding agencies’ response to the incident and the levels of anxiety, perceived risk and behaviour change. The importance of trust in determining public responses to public health crises has been noted in several previous studies.^11 15 20 21^ Trust is not necessarily a unitary construct and appears to be influenced by multiple factors.^21^ In our study we assessed several components, including perceived competence, expertise, benevolence, equity and access to sufficient resources. All of these showed room for improvement. Demonstrating that the official agencies can be trusted across all of these factors is likely to reduce public concern.

Several limitations should be borne in mind when considering our findings. In particular, whether the sample was representative of the population surrounding Salisbury is unknown. Although the response rate was low, the use of quotas for age and gender is likely to have mitigated selection bias, and while it is possible that the sample was biased towards people with a specific interest in the incident we attempted to reduce this by not revealing the full topic of the survey until after initial consent had been obtained. Nonetheless, the under-representation of people aged 18–44 in comparison with older age groups was notable. This may relate to a lower proportion within this group who own a landline telephone. Given that older age was associated with anxiety and anger, it could be that this bias has led us to overestimate the psychological impact of the incident. The number of analyses we conducted is a second limitation, raising the possibility of a type I error. Caution is required in interpreting some of our findings involving CIs that approached 1. A third limitation relates to the fact that the survey interviewed a sample from the general population of Salisbury and surrounding areas and did not focus specifically on those most affected by the incident. Care should be taken not to extrapolate our findings relating to the wider community to specific subsections within it, who may, for example, have had a qualitatively different set of concerns and who may have received more detailed information and support from official agencies.

Overall, the initial Salisbury incident appeared to have had a relatively modest impact on levels of emotion and risk perception, although the number who reported avoiding the city was notable. Emotional and behavioural responses were related to perceptions around the possible spread of contamination, the motives underlying the incident, the amount and quality of information received about the recovery and support efforts, and the levels of trust in government. As the residents of Salisbury have rightly attested, such responses should not be misconstrued as ‘panic’.^22^ In future incidents, demonstrating that contamination is known to be contained within specific areas, improving communication about any financial recovery package and promoting trust in responding agencies should all help in providing additional reassurance to the community.

## Supplementary Material

Reviewer comments

Author's manuscript
