# Supplementary material for: Public responses to the Salisbury Novichok incident: a cross-sectional survey of anxiety, anger, uncertainty, perceived risk and avoidance behaviour in the local community
Source: BMJ Open. 2020 Sep 25;10(9):e036071. doi: 10.1136/bmjopen-2019-036071 (PMC7520835; doi:10.1136/bmjopen-2019-036071)
Supplement: Supplementary data [file bmjopen-2019-036071supp001.pdf]

Salisbury Telephone Survey
Final topline results
(16-04-18)

- This topline presents the findings from a survey of residents living in Salisbury, Wiltshire, conducted by ICM Unlimited.
- ICM interviewed a representative sample of 500 adults aged 18+ living in Salisbury using a telephone (CATI) methodology. The interviewing took place between 5th and 13th April 2018.
- To ensure a broadly representative sample of Salisbury residents aged 18+, quotas were set by age and gender. Data have also been weighted to the same variables.
- A sample size of 500 produces data accurate to plus or minus four (+/-4) percentage points at the 95 per cent confidence level.
- Results are based on all respondents (500) unless otherwise stated.
- An asterisk (*) denotes a value that is greater than zero but less than one.
- Where results do not sum to 100%, this may be due to multiple responses, computer rounding or the exclusion of don't knows/not stated.
- Conducted in accordance with ISO 20252 and ISO 27001, the international standards for market research and information security management.

Rubin GJ, *et al. BMJ Open* 2020; 10:e036071. doi: 10.1136/bmjopen-2019-036071

Q2. Thinking about the events in Salisbury since 4th March 2018, how much have you seen, heard or read about what actually happened? SINGLE CODE

	%
A lot	89
A little	11
Nothing at all	*
Don't know	*

Q3. I'd like to ask you some questions about how you have been feeling over the past week in relation to the incident in Salisbury. For each of the following phrases, please tell me whether you have been feeling that way when thinking about the incident in Salisbury "Not at all, Somewhat, Moderately or Very Much". READ OUT EACH. SINGLE CODE FOR EACH OF THE 8 ITEMS BELOW

		Not at all	Somewhat	Moderately	Very much
**Calm**	%	7	14	27	52
**Tense**	%	75	12	11	3
**Upset**	%	57	17	17	9
**Relaxed**	%	10	16	33	41
**Content**	%	21	16	28	34
**Worried**	%	60	20	14	6
**Angry**	%	54	18	18	10
**Uncertain**	%	51	18	23	8

Q4. Following the incident, what is your biggest concern for the Salisbury and the community?

VERBATIMS PROVIDED

Q5. On a scale of 0 to 4, where 0 is not at all and 4 is a lot, to what degree do you feel your health is at risk as a consequence of the recent incident in Salisbury? SINGLE CODE

	%
0 - Not at all	74
1	14
2	7
3	4
4 - A lot	1
Don't know	1

Rubin GJ, *et al. BMJ Open* 2020; 10:e036071. doi: 10.1136/bmjopen-2019-036071

Q6. On Monday 5 March, several areas in Salisbury were cordoned off by the police as part of their investigations. These include the Mill Pub, Zizzi's restaurant, an area around a bench in The Maltings and the London Road cemetery. As far as you know, did you personally go to any of these areas between Sunday 4 March at 1:30pm and Monday 5 March at 11:30pm? MULTI CODE

	%
**Net: Yes**	**14**
Yes – General area around bench in The Maltings	8
Yes – Zizzi's restaurant	3
Yes – London Road Cemetery	2
Yes – Mill Pub	1
Yes – somewhere else	4
No	85
Not sure	1

Q7. Do you believe that you have been exposed to any of the chemical that was used in this incident? SINGLE CODE

	%
**Net: Yes**	*
Definitely yes	0
Probably yes	*
Not sure	3
Probably no	18
Definitely no	79
**Net: No**	**96**

Q8. Do you believe that any close family members or those dear to you have been exposed to any of the chemical that was used in this incident? SINGLE CODE

	%
**Net: Yes**	**3**
Definitely yes	1
Probably yes	3
Not sure	6
Probably no	21
Definitely no	70
**Net: No**	**91**

Q9. Have you sought help or advice about the effects of the incident in Salisbury on your own health? SINGLE CODE

	%
Yes	1
No	99

Rubin GJ, *et al. BMJ Open* 2020; 10:e036071. doi: 10.1136/bmjopen-2019-036071

Q10. I am going to read out a list of ways that the incident might have affected you. Please tell me if any of them apply to you. MULTICODE

	%
**Net: Have been affected**	**14**
There has been some other, serious, disruption to your day to day life	9
Your ability to go to work has been affected	4
You own a business that has been affected	2
Your income has been affected	2
None of the above	86

Q11. How much information have you seen, heard or read about the <u>recovery support</u>, for example the financial support package, the government has made available? SINGLE CODE

	%
A lot	18
A little	46
Nothing at all	32
Don't know	4

Q12. What measures / initiatives from the recovery support package have you seen, read or heard about?
Base: All respondents who are aware of the recovery support package (331).

VERBATIMS PROVIDED

Q13. To what extent do you believe that the information provided on the <u>recovery support package</u>:
READ OUT EACH. SINGLE CODE FOR EACH

Base: All respondents who are aware of the recovery support package (331)

		Very clear 5	4	Neither clear nor unclear 3	2	Very unclear 1	Don't know
**A) is clear**	%	15	16	32	14	9	14
		Very timely 5	4	Neither timely nor tardy 3	2	Tardy 1	Don't know
**B) is timely**	%	16	17	38	12	9	8
		Sufficient 5	4	Neither sufficient nor insufficient 3	2	Insufficient 1	Don't know
**C) is sufficient**	%	16	12	32	10	13	17
		Very useful 5	4	Neither useful nor not useful 3	2	Not useful 1	Don't know
**D) is useful**	%	24	23	30	5	8	10

Rubin GJ, *et al. BMJ Open* 2020; 10:e036071. doi: 10.1136/bmjopen-2019-036071

Q14. Where would you go to get information about the recovery support package?

Base: All respondents who are aware of the recovery support package (331).

VERBATIMS PROVIDED

Q15. To what extent do you agree or disagree with the following statements. Please answer strongly agree, agree, neither agree nor disagree, disagree, or strongly disagree. READ OUT EACH. SINGLE CODE FOR EACH

		Strongly agree	Agree	Neither	Disagree	Strongly disagree	Don't know	**Net: Agree**	**Net: Disagree**
**I think that sufficient support is being offered by the government to individuals directly affected**	%	8	30	32	10	6	13	**38**	**16**
**I think that sufficient support is being offered by the government to businesses directly affected**	%	6	30	24	15	7	17	**37**	**22**
**I think that sufficient support is being offered by the government to local residents across Salisbury**	%	7	34	27	12	7	13	**41**	**19**
**I think that sufficient support is being offered by the government to local businesses across Salisbury**	%	7	32	22	18	6	16	**38**	**24**

Q16. How much information have you seen, heard or read about the clean-up / decontamination process in Salisbury? SINGLE CODE

	%
A lot	32
A little	52
Nothing at all	14
Don't know	2

Rubin GJ, *et al. BMJ Open* 2020; 10:e036071. doi: 10.1136/bmjopen-2019-036071

ICM UNLIMITED

Q17. How long would you expect the clean-up process to last? SINGLE CODE. DO NOT READ OUT OUT OPTIONS

	%
Less than a month	22
One to two months	26
Between two to six months	19
Between six months to a year	5
More than a year	1
Don't know	26

Q18. Which of these following phrases do you think best describes recent events? READ OUT. REVERSE ORDER, SINGLE CODE

	%
An incident intended to harm only a single person	54
An incident intended to harm a small number of specific people	36
An incident intended to harm the wider public	6
Don't know	4

Q19. Since the incident in Salisbury, have you…? READ OUT EACH. SINGLE CODE FOR EACH

		Yes	No	Not applicable
**Deliberately reduced the amount you go into Salisbury**	%	19	80	1
**Washed or disposed of clothing or other items, specifically because they might have been contaminated with the chemical**	%	3	95	2
**Taken any other action to protect your health, or the health of a close family member or someone dear to you**	%	5	95	1
IF YES TO 3. And what is that that you have done?	%	VERBATIMS PROVIDED		

ICM UNLIMITED

Q20. The next set of statements are about how government and official agencies are dealing with issues relating to the incident in Salisbury (including public safety, clean-up process and recovery support).

I'd like you to tell me whether each one is probably true, probably false or if you're not sure. READ OUT EACH. SINGLE CODE FOR EACH

		Probably true	Probably false	Not sure
**In general, I think the government / official agencies are doing a good job of dealing with the incident**	%	76	9	15
**In general, I think the government / official agencies have enough resources to cope with the incident**	%	65	17	17
**In general, I think the government / official agencies have the necessary knowledge to deal with the incident**	%	74	13	13
**In general, I think the government / official agencies are acting in the public's best interests in dealing with the incident**	%	79	10	11
**In general, I think the government / official agencies are managing the incident in a fair way**	%	75	12	13
**In general, I feel confident in the government / official agencies ability to deal with the incident**	%	73	13	14

Rubin GJ, *et al. BMJ Open* 2020; 10:e036071. doi: 10.1136/bmjopen-2019-036071
