# Supplementary material for: Public responses to the Salisbury Novichok incident: a cross-sectional survey of anxiety, anger, uncertainty, perceived risk and avoidance behaviour in the local community
Source: BMJ Open. 2020 Sep 25;10(9):e036071. doi: 10.1136/bmjopen-2019-036071 (PMC7520835; doi:10.1136/bmjopen-2019-036071)
Supplement: Author's manuscript [file bmjopen-2019-036071.draft_revisions.pdf]

BMJ Open is committed to open peer review. As part of this commitment we make the peer review history of every article we publish publicly available.

When an article is published we post the peer reviewers' comments and the authors' responses online. We also post the versions of the paper that were used during peer review. These are the versions that the peer review comments apply to.

The versions of the paper that follow are the versions that were submitted during the peer review process. They are not the versions of record or the final published versions. They should not be cited or distributed as the published version of this manuscript.

BMJ Open is an open access journal and the full, final, typeset and author-corrected version of record of the manuscript is available on our site with no access controls, subscription charges or pay-per-view fees (http://bmjopen.bmj.com).

If you have any questions on BMJ Open's open peer review process please email info.bmjopen@bmj.com

BMJ Open

Public responses to the Salisbury Novichok incident: A cross-sectional survey of anxiety, anger, uncertainty, perceived risk and avoidance behaviour in the local community

Journal:	*BMJ Open*
Manuscript ID	bmjopen-2019-036071
Article Type:	Original research
Date Submitted by the Author:	28-Nov-2019

Complete List of Authors:	Rubin, GJ; King's College London, Institute of Psychiatry, Psychology and Neuroscience<br>Webster, Rebecca; King's College London, Institute of Psychiatry, Psychology and Neuroscience<br>Amlot, Richard; Public Health England Porton, Emergency Response Department<br>Carter, Holly; Public Health England Porton, Emergency Response Department<br>Weston, Dale; Public Health England Porton, Emergency Response Department<br>Wessely, Simon; King's College London, Institute of Psychiatry, Psychology and Neuroscience

Keywords:	PUBLIC HEALTH, Risk management < HEALTH SERVICES ADMINISTRATION & MANAGEMENT, TOXICOLOGY

SCHOLARONE™ Manuscripts

I, the Submitting Author has the right to grant and does grant on behalf of all authors of the Work (as defined in the below author licence), an exclusive licence and/or a non-exclusive licence for contributions from authors who are: i) UK Crown employees; ii) where BMJ has agreed a CC-BY licence shall apply, and/or iii) in accordance with the terms applicable for US Federal Government officers or employees acting as part of their official duties; on a worldwide, perpetual, irrevocable, royalty-free basis to BMJ Publishing Group Ltd ("BMJ") its licensees and where the relevant Journal is co-owned by BMJ to the co-owners of the Journal, to publish the Work in this journal and any other BMJ products and to exploit all rights, as set out in our licence.

The Submitting Author accepts and understands that any supply made under these terms is made by BMJ to the Submitting Author unless you are acting as an employee on behalf of your employer or a postgraduate student of an affiliated institution which is paying any applicable article publishing charge ("APC") for Open Access articles. Where the Submitting Author wishes to make the Work available on an Open Access basis (and intends to pay the relevant APC), the terms of reuse of such Open Access shall be governed by a Creative Commons licence – details of these licences and which Creative Commons licence will apply to this Work are set out in our licence referred to above.

Other than as permitted in any relevant BMJ Author's Self Archiving Policies, I confirm this Work has not been accepted for publication elsewhere, is not being considered for publication elsewhere and does not duplicate material already published. I confirm all authors consent to publication of this Work and authorise the granting of this licence.

For peer review only

DRAFT – NOT FOR CIRCULATION

Public responses to the Salisbury Novichok incident: A cross-sectional survey of anxiety, anger, uncertainty, perceived risk and avoidance behaviour in the local community

G James Rubin[1] (0000-0002-4440-0570), Rebecca K Webster[1] (0000-0002-5136-1098), Richard Amlôt[2], Holly Carter[2], Dale Weston[2], Simon Wessely[1] (0000-0002-6743-9929)

Affiliations

1: King's College London, Institute of Psychiatry, Psychology & Neuroscience, UK

2: Emergency Response Department, Public Health England, UK

Addresses

G James Rubin: King's College London, Institute of Psychiatry, Psychology and Neuroscience, Department of Psychological Medicine, Weston Education Centre, Cutcombe Road, London, SE5 9RJ, UK

Reader in the Psychology of Emerging Health Risks

Rebecca K Webster: King's College London, Institute of Psychiatry, Psychology and Neuroscience, Department of Psychological Medicine, Weston Education Centre, Cutcombe Road, London, SE5 9RJ, UK

Post-Doctoral Research Associate

Richard Amlôt: Emergency Response Department, Public Health England, Manor Farm Road, Porton Down, Salisbury, SP4 0JG

Professor of Practice in the Psychology of Health Protection

Dale Weston: Emergency Response Department, Public Health England, Manor Farm Road, Porton Down, Salisbury, SP4 0JG

Research Fellow

For peer review only

For peer review only - http://bmjopen.bmj.com/site/about/guidelines.xhtml

DRAFT – NOT FOR CIRCULATION

Holly Carter: Emergency Response Department, Public Health England, Manor Farm Road, Porton Down, Salisbury, SP4 0JG

Research Fellow

Simon Wessely: King's College London, Institute of Psychiatry, Psychology and Neuroscience, Department of Psychological Medicine, Weston Education Centre, Cutcombe Road, London, SE5 9RJ, UK

Regius Professor of Psychiatry

Corresponding author

James Rubin: gideon.rubin@kcl.ac.uk

For peer review only

For peer review only - http://bmjopen.bmj.com/site/about/guidelines.xhtml

DRAFT – NOT FOR CIRCULATION

Abstract

Objectives: Malicious incidents involving chemical agents sometimes trigger high public concern. We aimed to: 1) identify levels of emotion, perceived risk and behaviour change with regards to visiting Salisbury, one month after three people were poisoned with a nerve agent; and 2) test whether factors including receipt of information, beliefs about personal exposure and trust in government were associated with these outcomes.

Design: A cross-sectional telephone survey of a random sample of Salisbury residents.

Setting: Conducted between 5th and 13th April 2018.

Participants: 500 residents aged 18 or over.

Outcome measures: Self-reported anxiety, anger, uncertainty, perceived risk to self and avoidance of Salisbury.

Results: Any degree of anxiety, anger and uncertainty was reported by 40.6%, 29.8% and 30.6% of participants, respectively. For the majority, the level of emotion reported was mild. Only 7.0% met the criteria for high anxiety and 5.2% reported feeling any risk to their health, whereas 18.6% reported avoiding Salisbury. Factors associated with avoidance of Salisbury included being female, unable to rule out exposure for oneself, or of loved ones, believing the incident was targeted against the general public, and lower trust in the government and responding agencies. Hearing a lot or a little about the recovery support (e.g. financial packages) as opposed to nothing at all, and being satisfied with this information were associated with reduced avoidance.

Conclusions: Although the March 2018 Salisbury incident had a relatively modest impact on emotion and risk perception in the community, the number who reported avoiding the city was notable. In this, and future incidents, assuring people that contamination resulted from a targeted, rather than indiscriminate, incident; demonstrating that contamination is contained within specific areas; improving communication about any financial support; and promoting trust in responding agencies should help provide additional reassurance to the community.

For peer review only

DRAFT – NOT FOR CIRCULATION

Strengths and limitations of the study

- The study sample was demographically representative of adult residents living in Salisbury, UK.
- The study took place shortly after the Salisbury Novichok incident, and data collection took place over a short period of time.
- While it is possible that the sample was biased towards people with a specific interest in the incident, we attempted to reduce this by not revealing the full topic of the survey until after initial consent had been obtained.
- This study replicated previous findings about the importance of the perceived extensiveness of contamination and the perceived motivation for an incident in determining emotional and behavioural responses and risk perception.
- The response rate was extremely low, and although quota sampling may have mitigated the effects of this, whether the surveys are psychologically representative of the general population is unclear.

For peer review only

Introduction

Public health incidents involving chemical, biological, radiological or nuclear (CBRN) agents can sometimes trigger seemingly disproportionate levels of distress among the affected community. Previous incidents have witnessed high levels of anxiety or anger [1], spontaneous evacuations or attempts to avoid potentially contaminated areas [2 3], stigmatisation of people seen as contaminated [4 5], high levels of help-seeking among people who have not been exposed to the agent [6], and economic and social upheavals [7].

Attempts by responding agencies to reduce these effects often focus on improving communication with an affected community by identifying and addressing specific concerns (e.g.[8 9]) and by building trust with those affected (e.g. [10 11]). Research in this area has resulted in generic guidelines that can be used to assist in the acute phase of these responses, for example by identifying topics that members of the public are likely to have particular misgivings about, such as perceived exposure to a toxic substance [12 13]. Less research has focussed on the importance of other factors that may become relevant once the acute phase of a CBRN incident has passed. For example, the importance of communication about the clean-up operation or the level of public satisfaction with any financial support that is subsequently made available.

The literature on CBRN incidents is also problematic because it often relies on the use of hypothetical scenarios, rather than the study of actual incidents. For example, in a 2012 systematic review of communication research relating to the deliberate use of CBRN material [12], out of the 33 studies identified, only 12 investigated genuine incidents, 11 of which related to the US anthrax attacks of 2001. The twelfth explored the reactions of the London population to the use of radioactive Polonium 210 in the apparent assassination of the former Russian intelligence officer Alexander Litvinenko, an incident which resulted in a major public health response after a restaurant and hotel bar were found to be contaminated [9]. The study found that increased concern among members of the public was linked to the perceived motivation underlying the incident (i.e. that it had been targeted at the wider public rather than at a single individual), and to a belief that contamination might occur away from the areas that had been cordoned-off by the emergency services.

While studies on genuine incidents involving the use of CBRN agents are rare, rarer still are studies that are able to reproduce previous findings. The idiosyncrasies of each incident in terms of the population affected, public familiarity with the agent and the media narrative

For peer review only - http://bmjopen.bmj.com/site/about/guidelines.xhtml

surrounding the incident often make it difficult to compare results. But on 4 March 2018, an incident occurred in England which bore remarkable similarities to the Polonium 210 incident. Sergei Skripal, an ex-Russian intelligence officer, his daughter and a police officer became seriously ill following exposure to the nerve agent Novichok in what the UK Government described as an attempted assassination. The incident occurred in the small English city of Salisbury (population: 40,000), whose residents suddenly found themselves at the centre of a major police investigation, media furore and diplomatic crisis. Several areas of the city were cordoned off by police both to preserve forensic evidence and to prevent other members of the public from being exposed to the agent. Investigations initially focussed on a pub and restaurant that were visited by the Skripals shortly before they became ill.

A week after the cordons went up, the Chief Medical Officer for England advised that, as a precautionary measure, members of the public who had been in the pub or restaurant at the time of the incident should wash the clothes they had been wearing, wipe or wash personal items such as mobile phones or jewellery, and seal dry-clean only clothing in plastic bags. It was later announced that dry-clean only clothing would be collected and incinerated. As the clean-up operation continued, a package of financial measures was announced by the government to support individuals and businesses that had been affected by a decline in visitors into the city. It was not until 1 March 2019 that the decontamination efforts in Salisbury finally came to an end.

In this study, we used a telephone survey of the local population of Salisbury conducted one month after the incident began in order to: 1) identify levels of anxiety, anger, uncertainty, perceived risk and changes in behaviour regarding visiting Salisbury; 2) test whether factors that may be particularly relevant in the acute stage of the incident (amount heard about the incident, perceived exposure of self, perceived exposure of significant others, perceived motivation for the incident) were associated with these outcomes and 3) test whether factors related to the recovery stage of the incident were associated with these outcomes, specifically the amount heard about the clean-up or recovery effort, the satisfaction with this information or the level of support offered, the expected length of the clean-up, the level of impact on the participant's daily lives or the level of trust in the government and responding agencies.

For peer review only - http://bmjopen.bmj.com/site/about/guidelines.xhtml

DRAFT – NOT FOR CIRCULATION

Methods

Design

Between 5th and 13th April 2018, ICMUnlimited conducted a telephone survey of 500 residents living in Salisbury, UK. This sample size provided a total 95% confidence interval of approximately plus or minus 4% for each prevalence estimate. To achieve a broadly representative sample, ICMUnlimited used a random approach to ensure that all landline telephone numbers within the geographical region had an equal chance of being called, while quotas were set for the sample based on age and gender based on Census 2011 data. To reduce selection bias, participants were initially informed that the survey related to “issues affecting the UK” and were only informed after providing consent that the particular issues of interest were the recent events in Salisbury. Interviews typically lasted around 10 minutes.

The study was approved by King’s College London’s Psychiatry, Nursing and Midwifery Research Ethics Subcommittee (reference: HR-17/18-5881).

Patient and public involvement

There was no formal patient or public involvement in this study.

Participants

In order to take part, respondents were required to be 18 years or older, to live in Salisbury and surrounding areas (postcode areas SP1, SP2, SP3, SP4, SP5, SP6) and to speak English.

Outcomes

The supplemental material provides a copy of the wording used for all items in the survey.

Anxiety was measured using an adapted version of the six item state trait anxiety inventory (STAI-6) [14] which asked participants to report how they had been feeling over the past week “when thinking about the incident in Salisbury.” The STAI-6 provides scores from 6 (least anxiety) to 24 (most anxiety). In line with previous work on public responses to major incidents [1 15], we categorised people who scored 12 or more as having anxiety about the incident and those who scored 18 or more as having high anxiety. Two additional items were added to the end of the inventory to assess anger and uncertainty. These were analysed separately, with responses of ‘moderately’ or ‘very much’ being taken as indicating the presence of anger or uncertainty.

For peer review only - http://bmjopen.bmj.com/site/about/guidelines.xhtml

DRAFT – NOT FOR CIRCULATION

Perceived risk was measured using a single item adapted from previous work on the Polonium 210 incident [9] asking participants "to what degree do you feel your health is at risk as a consequence of the recent incident in Salisbury." Responses were given on a scale of 0 (not at all) to 4 (a lot). As with the Polonium 210 incident, we categorised people who gave a response of 3 or 4 as perceiving a personal health risk.

Changes in behaviour regarding visiting Salisbury was assessed using the item: "Since the incident in Salisbury, have you deliberately reduced the amount you go into Salisbury?" Participants could respond "yes" or "no".

Predictor measures related to the acute stage of the incident

Participants were asked how much they had seen, heard or read about the incident, with response options being a lot, a little and nothing at all.

In order to assess perceived exposure, participants were asked whether they believed that they, or a close family member or someone dear to them, had been exposed to "any of the chemical that was used in this incident" (definitely yes, probably yes, not sure, probably no, definitely no).

In order to assess perceptions about the motives underlying the incident, we adapted a single item from the Polonium 210 study [9] which asked participants whether they thought the incident was intended to harm only a single person, a small number of people, or the wider public.

Predictor measures related to the recovery stage

Participants were asked how much they had seen, heard or read about the clean-up process in Salisbury and about recovery support "for example the financial support package the government has made available." For both questions, possible responses were a lot, a little and nothing at all. For those aware of the recovery support package, follow-up questions asked the extent to which they felt the information they had received was: clear; timely; sufficient; and useful. These items were summed to give a scale with acceptable internal reliability (Cronbach's alpha 0.78), from 4 (least satisfied) to 20 (most satisfied). Participants were also asked their level of agreement with statements that sufficient support was being offered by the government to: individuals directly affected, businesses directly affected, local residents across Salisbury and local business across Salisbury. Again, these items were

For peer review only - http://bmjopen.bmj.com/site/about/guidelines.xhtml

summed to give a scale with acceptable internal reliability (Cronbach's alpha 0.88) from 4 (least satisfied) to 20 (most satisfied).

Participants were asked a single open-ended item on their expectations for the length of the clean-up process.

Impact on the participant's financial or daily life was assessed by asking whether "your ability to go to work has been affected," "you own a business that has been affected," "your income has been affected," or "there has been some other, serious, disruption to your day to day life."

Trust in the official response was assessed using six items developed for use in an infectious disease outbreak [16] which assessed whether, in relation to the incident, the participant thought the government and official agencies were doing a good job, had enough resources, had the necessary knowledge, were acting in the public's best interests, were managing the incident in a fair way, and whether they felt confident about their ability to deal with the incident. Responses to these items showed acceptable reliability (Cronbach's alpha 0.74) and were summed to provide a score of 6 (least trust) to 18 (most trust), and then dichotomised into scores of low (12 or below) versus high (13 or more) trust.

Statistical analysis

For all analyses we coded responses of "don't know" or "not applicable" as missing data.

We used logistic regressions to calculate adjusted odds ratios to test the association between gender and age (controlled for each other) and each of the outcome variables, as well as for each predictor measure (controlling for gender and age) and each outcome variable.

For perceived exposure variables, we compared participants who believed they had probably or definitely been exposed or who were not sure, against those who believed they had probably or definitely not been exposed. For perceived impact, we compared those who reported impact on any aspect of their life with those who reported no impact.

For ease of interpretation, all analyses were conducted using unweighted data.

For peer review only - http://bmjopen.bmj.com/site/about/guidelines.xhtml

DRAFT – NOT FOR CIRCULATION

Results

Response rates

In order to obtain 500 valid responses, ICMUnlimited made a total of 18,753 calls, resulting in contact with 6,808 people who were within quota (7.3%). Low response rates are typical of this style of telephone survey, and are not necessarily indicative of high response bias [17]. Two hundred and eighty participants (56.0%) were female and 220 (44.0%) male. One hundred and sixty-eight participants (33.6%) were aged 18 to 44, 195 (39.0%) were aged 45 to 64, and 137 (27.4%) were 65 or over. Seventeen participants (3%) reported having been in the pub or restaurant at the centre of incident.

Main outcomes

The main outcomes are summarised in Table 1. Two hundred and three participants (40.6%) were defined as having some level of anxiety, while anger and uncertainty were reported by 149 (29.8%) and 153 (30.6%) participants, respectively. Only 35 (7.0%) participants met the criteria for high anxiety and only 26 (5.2%) reported feeling that there was any risk to their own health. Ninety-two (18.6%) reported having deliberately reduced the amount they go into Salisbury.

Acute predictors of outcomes

Table 2 shows the associations between the various acute stage predictor variables and emotional response. In general, older adults were more likely to report anxiety and anger, while women were more likely to report anxiety. People who were unable to rule out the possibility that they themselves had been exposed to the chemical agent were more likely to report anxiety, anger and uncertainty, while people who were unable to rule out exposure amongst friends or relatives were more likely to report anxiety and uncertainty. Those who believed the incident was targeted at the wider public were more likely to report uncertainty than people who felt it was targeted at a single individual.

Although the small number of people categorised as having high anxiety prevented us from conducting detailed statistical analyses, it was notable that most people in this group had not been to a contaminated location and reported believing that neither they nor any close friends or relatives had been exposed to the nerve agent.

For peer review only - http://bmjopen.bmj.com/site/about/guidelines.xhtml

Table 3 shows the association between the acute-stage predictors and perceived risk to self or behaviour change. People who could not rule out that they themselves had been exposed to the chemical agent were more likely to feel that their health was at risk and to have avoided visiting Salisbury. The same pattern of findings was also true for those who could not rule out exposure of friends or family members, and those who felt the incident was targeted at the wider public rather than at a single person. Being female was only associated with avoidance of Salisbury, whereas participants who had heard little or no information about the incident were more likely to feel that their health was at risk.

Recovery stage predictors of outcomes

Greater satisfaction with the information given out about the recovery support was associated with lower levels of anxiety and uncertainty, while participants who reported having heard nothing about the recovery support or who were less satisfied with the support available had a greater likelihood of avoiding Salisbury. Those who thought the clean-up would take less than one month were more likely to perceive a lower risk to self. Lower trust in the government and official agencies was associated with a greater likelihood of anxiety, uncertainty, perceived risk to self and avoiding Salisbury.

For peer review only

DRAFT – NOT FOR CIRCULATION

Discussion

One month following the Novichok incident in Salisbury, many members of the local community reported some degree of anxiety (40.6%), anger (29.8%) or uncertainty (30.6%). By way of comparison, using the same scale, 23.7% of the British population reported some anxiety at the very earliest stages of the swine flu outbreak [15]. The number reporting high anxiety was relatively low, however, at 7.0%. Again, for comparison, the number reporting high anxiety among the general public of Great Britain during the early stages of the swine flu outbreak was 2.1% [15], while amongst British nationals in Japan at the time of the Fukushima nuclear disaster it was 29.7% [1]. Levels of perceived risk were also low (5.2%) and slightly lower than those found during the Polonium 210 incident (11.7% [18]). Despite emotional responses being relatively low however, behavioural change was apparent, with 18.6% of people from the local area reporting deliberately reducing the amount they went into Salisbury. This is similar to the 20% of Londoners who reported intending to going into central London less often after the 7/7 bombings [19].

Our analyses suggested that there were three core issues underlying these findings. First, a pattern of associations was identified between the outcomes and believing that exposure might have occurred to oneself or a loved one, or feeling that the incident had been targeted at the wider public. Tellingly, most people reporting high anxiety had not been present within an area that was later cordoned off. Similar findings were also observed during the Polonium 210 incident [18] and suggest a level of concern that contamination may deliberately or accidentally occur beyond the areas identified by the responding agencies. Emphasising that this is unlikely should reduce levels of concern among the population, but responding agencies may need to exercise caution about this. In this incident, on 3 July 2018, two further members of the public became ill following accidental exposure to a source of Novichok from an location outside of the initial cordons. One subsequently died. As a result, multiple new locations were cordoned off by police, and members of the public were once again issued with advice about washing clothes and possessions.

The second core issue underlying concern related to the level of support being provided to the community during the recovery phase. The incident had implications for the economic wellbeing of the community, with local businesses and their employees affected by the presence of police cordons and a reduction in the number of visitors to the city. Although several measures were introduced to offset this, at the time of our survey most people had

For peer review only - http://bmjopen.bmj.com/site/about/guidelines.xhtml

heard little or nothing about them. This in turn appeared to underlie some of the anxiety and uncertainty reported by residents. Interestingly, an association was also found between having heard more about the recovery package or satisfaction with the support available, and being less likely to avoid Salisbury. Whether the information provided reduced concerns about visiting the city, or whether people who visited Salisbury were more likely to encounter information about the recovery effort is unclear. Nonetheless, ensuring that communication about recovery support is widely disseminated should be beneficial in future incidents.

The third core issue related to trust. Strong associations were observed between greater trust in the government and responding agencies' response to the incident and levels of anxiety, perceived risk and behaviour change. The importance of trust in determining public responses to public health crises has been noted in several previous studies [11 15 20 21]. Trust is not necessarily a unitary construct and appears to be influenced by multiple factors [21]. In our study we assessed several components, including perceived competence, expertise, benevolence, equity, and access to sufficient resources. All of these showed room for improvement. Demonstrating that the official agencies can be trusted across all of these factors is likely to reduce public concern.

Several limitations should be borne in mind when considering our findings. In particular, whether the sample was representative of the population surrounding Salisbury is unknown. Although the response rate was low, the use of quotas for age and gender is likely to have mitigated selection bias and while it is possible that the sample was biased towards people with a specific interest in the incident, we attempted to reduce this by not revealing the full topic of the survey until after initial consent had been obtained. The number of analyses we conducted is a second limitation, raising the possibility of a Type I error. Caution is required in interpreting some of our findings involving confidence intervals that approached one. A third limitation relates to the fact that the survey interviewed a sample from the general population of Salisbury and surrounding areas, and did not focus specifically on those most affected by the incident. Care should be taken not to extrapolate our findings relating to the wider community to specific subsections within it, who may, for example, have had a qualitatively different set of concerns and who may have received more detailed information and support from official agencies.

For peer review only - http://bmjopen.bmj.com/site/about/guidelines.xhtml

DRAFT – NOT FOR CIRCULATION

Overall, the initial Salisbury incident appeared to have had a relatively modest impact on levels of emotion, and risk perception, although the number who reported avoiding the city was notable. Emotional and behavioural responses were related to perceptions around the possible spread of contamination, the motives underlying the incident, the amount and quality of information received about the recovery and support efforts and levels of trust in government. As the residents of Salisbury have rightly attested, such responses should not be misconstrued as "panic."[22] In future incidents, demonstrating that contamination is known to be contained within specific areas, improving communication about any financial recovery package and promoting trust in responding agencies should all help in providing additional reassurance to the community.

For peer review only - http://bmjopen.bmj.com/site/about/guidelines.xhtml

DRAFT – NOT FOR CIRCULATION

Acknowledgements

We would like to thank the study participants for giving up their time to support this study.

Author contributions

All authors contributed equally to the conceptualisation and design of this study, and read and approved the final version of the manuscript. GJR and RKW conducted the data analysis, and GJR wrote the first draft of the manuscript with input from all other authors. GJR accepts full responsibility for the work and conduct of the study, had access to the data, and controlled the decision to publish. The corresponding author attests that all listed authors meet authorship criteria and that no others meeting the criteria have been omitted.

Role of study sponsor and funder

The sponsor and funder of the study had no role in study design, data collection, data analysis, data interpretation, or writing of the report. The corresponding author had full access to all the data in the study and had final responsibility for the decision to submit for publication.

Declaration of interests

Rubin has received a payment from the UK Government to provide advice about public responses to the Salisbury incident.

Financial disclosure statements

The research was part funded by the National Institute for Health Research Health Protection Research Units (NIHR HPRU) in Emergency Preparedness and Response at King's College London, and Modelling Methodology at Imperial College London, in partnership with Public Health England (PHE), and part funded by DEFRA.

The views expressed are those of the author(s) and not necessarily those of the NHS, the NIHR, the Department of Health, DEFRA or Public Health England.

Copyright statement

The Corresponding Author has the right to grant on behalf of all authors and does grant on behalf of all authors, a worldwide licence to the Publishers and its licensees in perpetuity, in all forms, formats and media (whether known now or created in the future), to i) publish,

For peer review only - http://bmjopen.bmj.com/site/about/guidelines.xhtml

reproduce, distribute, display and store the Contribution, ii) translate the Contribution into other languages, create adaptations, reprints, include within collections and create summaries, extracts and/or, abstracts of the Contribution, iii) create any other derivative work(s) based on the Contribution, iv) to exploit all subsidiary rights in the Contribution, v) the inclusion of electronic links from the Contribution to third party material where-ever it may be located; and, vi) licence any third party to do any or all of the above

Declaration of competing interests

All authors have completed the ICMJE uniform disclosure form at www.icmje.org/coi_disclosure.pdf and declare: all authors had financial support from the NIHR HPRU for the submitted work; no financial relationships with any organisations that might have an interest in the submitted work in the previous three years; no other relationships or activities that could appear to have influenced the submitted work.

Full access to data

All authors had full access to all of the data in the study and can take responsibility for the integrity of the data and the accuracy of the data analysis.

Transparency declaration

The lead author confirms that this manuscript is an honest, accurate and transparent account of the study being reported. No important aspects of the study have been omitted and any discrepancies from the study as planned have been explained.

Data sharing

No further data available.

For peer review only

DRAFT – NOT FOR CIRCULATION

References

1. Rubin GJ, Amlôt R, Wessely S, et al. Anxiety, distress and anger among British nationals in Japan following the Fukushima nuclear accident. *Br J Psychiatry* 2012;201(5):400-07. doi: 10.1192/bjp.bp.112.111575
2. Rubin GJ, Dickmann P. How to reduce the impact of "low-risk patients" following a bioterrorist incident: Lessons from SARS, anthrax, and pneumonic plague. *Biosecur Bioterrorism* 2010;8(1):37-43. doi: 10.1089/bsp.2009.0059
3. Ziegler DJ, Brunn SD, Johnson JH. Evacuation from a technological disaster. *Geogr Rev* 1981;71(1):1-16.
4. Person B, Sy F, Holton K, et al. Fear and stigma: The epidemic within the SARS outbreak. *Emerg Infect Dis* 2004;10(2):358-63.
5. Bromet EJ. Emotional consequences of nuclear power plant disasters. *Health Phys* 2014;106(2):206-10. doi: http://dx.doi.org/10.1097/HP.0000000000000012
6. Engel CC, Locke S, Reissman DB, et al. Terrorism, trauma and mass casualty triage: How might we solve the latest mind-body problem? *Biosecur Bioterrorism* 2007;5(2):155-63.
7. Collins DL, de Carvalho AB. Chronic stress from the Goiania 137Cs radiation accident. *Behav Med* 1993;18:149-57.
8. Rubin GJ, Potts HWW, Michie S. The impact of communications about swine flu (influenza A HINIv) on public responses to the outbreak: Results from 36 national telephone surveys in the UK. *Health Technol Assess* 2010;14(34):183-266. doi: 10.3310/hta14340-03
9. Rubin GJ, Page L, Morgan O, et al. Public information needs after the poisoning of Alexander Litvinenko with polonium-210 in London: Cross sectional telephone survey and qualitative analysis. *Br Med J* 2007;335(7630):1143-46. doi: 10.1136/bmj.39367.455243.BE
10. Peters RG. A study of the factors determining perceptions of trust and credibility in environmental risk communication: the importance of overcoming negative stereotypes. *Int Arch Occup Environ Health* 1996;68:442-47.
11. Meredith LS, Eisenman DP, Rhodes H, et al. Trust influences response to public health messages during a bioterrorist event. *J Health Commun* 2007;12:217-32.
12. Rubin GJ, Chowdhury AK, Amlôt R. How to communicate with the public about chemical, biological, radiological, or nuclear terrorism: A systematic review of the literature. *Biosecur Bioterrorism* 2012;10(4):383-95. doi: 10.1089/bsp.2012.0043
13. Hyer RH, Covello VT. Effective media communication during public health emergencies. Geneva: World Health Organisation 2005.
14. Marteau TM, Becker H. The development of a six-item short-form of the state scale of the Speilberger state-trait anxiety inventory (STAI). *Br J Clin Psychol* 1992;31:301-06.
15. Rubin GJ, Amlot R, Page L, et al. Public perceptions, anxiety, and behaviour change in relation to the swine flu outbreak: cross sectional telephone survey. *Br Med J* 2009;339:b2651. doi: http://dx.doi.org/10.1136/bmj.b2651
16. Rubin GJ, Bakhshi S, Amlot R, et al. The design of a survey questionnaire to measure perceptions and behaviour during an influenza pandemic: the Flu TElephone Survey Template (FluTEST). Southampton UK: Queen's Printer and Controller of HMSO 2014. This work was produced by Rubin et al. under the terms of a commissioning contract issued by the Secretary of State for Health. This issue may be freely reproduced for the purposes of private research and study and extracts (or indeed, the full report) may be included in professional journals provided that suitable acknowledgement is made and the reproduction is not associated with any form of

For peer review only - http://bmjopen.bmj.com/site/about/guidelines.xhtml

DRAFT – NOT FOR CIRCULATION

advertising. Applications for commercial reproduction should be addressed to: NIHR Journals Library, National Institute for Health Research, Evaluation, Trials and Studies Coordinating Centre, Alpha House, University of Southampton Science Park, Southampton SO16 7NS, UK. 2014.
17. O'Cathain A, Knowles E, Nicholl J. Testing survey methodology to measure patients' experiences and views of the emergency and urgent care system: Telephone versus postal survey. *BMC Med Res Methodol* 2010;10(52)
18. Rubin GJ, Amlôt R, Page L, et al. Assessing Perceptions about Hazardous Substances (PATHS): The PATHS questionnaire. *J Health Psychol* 2013;18(8):1100-13. doi: 10.1177/1359105312459096
19. Rubin GJ, Brewin CR, Greenberg N, et al. Psychological and behavioural reactions to the bombings in London on 7 July 2005: cross sectional survey of a representative sample of Londoners. *BMJ* 2005;331(7517):606. doi: 10.1136/bmj.38583.728484.3A
20. Tang CSK, Wong C-Y. An outbreak of the severe acute respiratory syndrome: predictors of health behaviors and effect of community prevention measures in Hong Kong, China. *Am J Public Health* 2003;93(11):1887-88.
21. Wray R, Rivers J, Whitworth A, et al. Public perceptions about trust in emergency risk communication: Qualitative research findings. *Int J Mass Emerg Disasters* 2006;24(1):45-75.
22. Morris S, Bannock C. 'The snow caused more panic': Salisbury shrugs off attack. *The Guardian* 2018.

For peer review only

DRAFT – NOT FOR CIRCULATION

Table 1: Emotional and behavioural reactions to the Salisbury poisoning

**Emotional or behavioural outcome**	**Number reporting that outcome / number with valid response (%)**
High or moderate anxiety	203 / 500 (40.6%)
High anxiety	35 / 500 (7.0%)
Anger	149 / 500 (29.8%)
Uncertainty	153 / 500 (30.6%)
Perceived personal risk	26 / 498 (5.2%)
Reduced amount you go into Salisbury	92 / 494 (18.6%)

For peer review only

For peer review only - http://bmjopen.bmj.com/site/about/guidelines.xhtml

DRAFT – NOT FOR CIRCULATION

Table 2: The association between acute stage predictor measures and emotional outcomes (odds ratios are adjusted for age and gender)

Variable	Variable levels	No (%) of participants	No (%) reporting anxiety	Odds ratio (95% confidence interval)	No (%) reporting anger	Odds ratio (95% confidence interval)	No (%) reporting uncertainty	Odds ratio (95% confidence interval)
Age	65+	137 (27.4%)	72 (52.6%)	**2.2 (1.4 to 3.5)**	65 (40.9%)	**2.6 (1.5 to 4.2)**	46 (33.6%)	1.5 (0.9 to 2.5)
	45-64	195 (39.0%)	72 (36.9%)	1.1 (0.7 to 1.7)	57 (29.2%)	1.5 (0.9 to 2.4)	64 (32.8%)	1.4 (0.9 to 2.2)
	18-44	168 (33.6%)	59 (35.1%)	Reference	36 (21.4%)	Reference	43 (25.6%)	Reference
Gender	Female	280 (56.0%)	129 (46.1%)	**1.8 (1.3 to 2.6)**	84 (30.0%)	1.1 (0.7 to 1.6)	89 (31.8%)	1.2 (0.8 to 17)
	Male	220 (44.0%)	74 (33.6%)	Reference	65 (29.5%)	Reference	64 (29.1%)	Reference
How much heard	A lot	449 (90.2%)	180 (40.1%)	0.8 (0.5 to 1.5)	129 (28.7%)	0.7 (0.3 to 1.2)	140 (31.2%)	1.6 (0.8 to 3.2)
	A little / nothing	49 (9.8%)	23 (46.9%)	Reference	19 (38.8%)	Reference	11 (22.4%)	Reference
Belief in exposure (self)	Definitely, probably or not sure	21 (4.2%)	16 (76.2%)	**5.5 (2.0 to 15.7)**	11 (52.4%)	**2.9 (1.2 to 7.0)**	14 (66.7%)	**4.9 (1.9 to 12.4)**
	Definitely or probably not	479 (95.8%)	187 (39.0%)	Reference	138 (28.8%)	Reference	139 (29.0%)	Reference
Belief in exposure (others)	Definitely, probably or not sure	44 (8.8%)	27 (61.4%)	**2.6 (1.3 to 4.9)**	18 (40.9%)	1.8 (0.96 to 3.5)	26 (59.1%)	**3.8 (2.0 to 7.2)**
	Definitely or probably not	456 (91.2%)	176 (38.6%)	Reference	131 (28.7%)	Reference	127 (27.9%)	Reference
Incident intended to harm…	Wider public	31 (6.2%)	18 (58.1%)	2.1 (0.96 to 4.5)	13 (41.9%)	1.5 (0.7 to 3.2)	16 (51.6%)	**2.5 (1.2 to 5.3)**
	Small number of specific people	187 (37.4%)	80 (42.8%)	1.2 (0.8 to 1.8)	53 (28.3%)	0.9 (0.6 to 1.4)	56 (29.9%)	1.0 (0.7 to 1.5)
	A single person	265 (54.9%)	101 (38.1%)	Reference	81 (30.6%)	Reference	78 (29.4%)	Reference

For peer review only - http://bmjopen.bmj.com/site/about/guidelines.xhtml

DRAFT – NOT FOR CIRCULATION

Table 3: The association between acute stage predictor measures, risk perception and behaviour (odds ratios are adjusted for age and gender

Variable	Variable levels	No (%) of participants	No (%) reporting personal risk perception	Odds ratio (95% confidence interval)	No (%) reporting avoiding Salisbury	Odds ratio (95% confidence interval)
Age	65+	137 (27.4%)	9 (6.6%)	1.9 (0.7 to 5.6)	20 (15.2%)	0.7 (0.4 to 1.2)
	45-64	195 (39.0%)	11 (5.7%)	1.6 (0.6 to 4.4)	35 (17.9%)	0.8 (0.5 to 1.3)
	18-44	168 (33.6%)	6 (3.6%)	Reference	37 (22.2%)	Reference
Gender	Female	280 (56.0%)	16 (5.7%)	1.3 (0.6 to 3.0)	61 (22.1%)	**1.7 (1.1 to 2.7)**
	Male	220 (44.0%)	10 (4.6%)	Reference	31 (14.2%)	Reference
How much heard	A lot	449 (90.2%)	20 (4.5%)	**0.3 (0.1 to 0.9)**	84 (18.8%)	1.2 (0.5 to 2.7)
	A little / nothing	49 (9.8%)	6 (12.2%)	Reference	8 (17.4%)	Reference
Belief in exposure (self)	Definitely, probably or not sure	21 (4.2%)	6 (28.6%)	**9.3 (3.2 to 26.9)**	9 (42.9%)	**3.5 (1.4 to 8.8)**
	Definitely or probably not	479 (95.8%)	20 (4.2%)	Reference	83 (17.5%)	Reference
Belief in exposure (others)	Definitely, probably or not sure	44 (8.8%)	7 (15.9%)	**4.4 (1.7 to 11.4)**	19 (45.2%)	**4.0 (2.0 to 7.7)**
	Definitely or probably not	456 (91.2%)	19 (4.2%)	Reference	73 (16.2%)	Reference
Incident intended to harm…	Wider public	31 (6.2%)	7 (22.6%)	**7.8 (2.6 to 22.9)**	13 (44.8%)	**5.0 (2.2 to 11.3)**
	Small number of specific people	187 (37.4%)	8 (4.3%)	1.2 (0.5 to 3.4)	37 (20.2%)	1.4 (0.9 to 2.3)
	A single person	265 (54.9%)	9 (3.4%)	Reference	40 (15.1%)	Reference

For peer review only - http://bmjopen.bmj.com/site/about/guidelines.xhtml

DRAFT – NOT FOR CIRCULATION

Table 4: The association between recovery predictor measures and emotional outcomes (odds ratios are adjusted for age and gender)

Variable	Variable levels	No (%) of participants	No (%) reporting anxiety	Odds ratio (95% confidence interval)	No (%) reporting anger	Odds ratio (95% confidence interval)	No (%) reporting uncertainty	Odds ratio (95% confidence interval)
How much heard - clean-up	A lot	169 (34.3%)	75 (44.4%)	0.9 (0.5 to 1.5)	56 (33.1%)	1.3 (0.7 to 2.5)	56 (33.1%)	1.3 (0.7 to 2.6)
	A little	257 (52.2%)	95 (37.0%)	0.7 (0.4 to 1.2)	73 (28.4%)	1.2 (0.6 to 2.2)	78 (30.4%)	1.2 (0.7 to 2.3)
	Nothing	66 (13.4%)	30 (45.5%)	Reference	17 (25.8%)	Reference	17 (25.8%)	Reference
How much heard - recovery	A lot	96 (20.0%)	35 (36.5%)	**0.5 (0.3 to 0.9)**	30 (31.3%)	0.9 (0.5 to 1.5)	25 (26.0%)	0.6 (0.3 to 1.04)
	A little	235 (49.0%)	93 (39.6%)	0.7 (0.4 to 1.03)	70 (29.8%)	0.9 (0.5 to 1.4)	72 (30.6%)	0.8 (0.5 to 1.2)
	Nothing	149 (31.0%)	69 (46.3%)	Reference	45 (30.2%)	Reference	52 (34.9%)	Reference
Satisfaction with recovery information	Scale of 4 (least) to 20 (most)	Mean score: 13.2 (sd 3.8)	Not applicable	1.0 (0.9 to 1.04)	Not applicable	0.9 (0.9 to 1.002)	Not applicable	1.0 (0.9 to 1.07)
Satisfaction with support offered	Scale of 4 (least) to 20 (most)	Mean score: 12.9 (sd 3.4)	Not applicable	**0.9 (0.9 to 0.99)**	Not applicable	1.0 (0.9 to 1.02)	Not applicable	**0.9 (0.9 to 0.98)**
Expected length of clean-up	Less than a month	108 (29.9%)	39 (36.1%)	0.8 (0.5 to 1.4)	28 (25.9%)	0.9 (0.5 to 1.5)	40 (37.0%)	1.6 (0.9 to 2.8)
	One to two months	124 (34.3%)	50 (40.3%)	1.0 (0.6 to 1.6)	40 (32.3%)	1.2 (0.7 to 2.1)	39 (31.5%)	1.3 (0.8 to 2.2)
	Two months or more	129 (35.7%)	52 (40.3%)	Reference	37 (28.7%)	Reference	34 (26.4%)	Reference
Impact on daily life	Some impact	68 (13.6%)	33 (48.5%)	1.5 (0.9 to 2.6)	23 (33.8%)	1.3 (0.7 to 2.2)	25 (36.8%)	1.4 (0.8 to 2.4)
	No impact	432 (86.4%)	170 (39.4%)	Reference	126 (29.2%)	Reference	128 (29.6%)	Reference
Trust in government	Low trust	67 (13.4%)	35 (52.2%)	**1.8 (1.04 to 3.0)**	23 (34.3%)	1.3 (0.8 to 2.3)	24 (35.8%)	1.3 (0.8 to 2.3)
	High trust	433 (86.6%)	168 (38.8%)	Reference	126 (29.1%)	Reference	129 (29.8%)	Reference

For peer review only - http://bmjopen.bmj.com/site/about/guidelines.xhtml

DRAFT – NOT FOR CIRCULATION

Table 5: The association between recovery predictor measures, risk perception and behaviour (odds ratios are adjusted for age and gender

Variable	Variable levels	No (%) of participants	No (%) reporting personal risk perception	Odds ratio (95% confidence interval)	No (%) reporting avoiding Salisbury	Odds ratio (95% confidence interval)
How much heard - clean-up	A lot	169 (34.3%)	9 (5.3%)	1.1 (0.3 to 4.1)	32 (19.2%)	0.8 (0.4 to 1.6)
	A little	257 (52.2%)	13 (5.1%)	1.1 (0.3 to 3.9)	45 (17.6%)	0.7 (0.4 to 1.4)
	Nothing	66 (13.4%)	3 (4.6%)	Reference	15 (23.1%)	Reference
How much heard - recovery	A lot	96 (20.0%)	3 (3.1%)	0.3 (0.1 to 1.1)	12 (12.5%)	**0.4 (0.2 to 0.8)**
	A little	235 (49.0%)	11 (4.7%)	0.5 (0.2 to 1.1)	35 (15.2%)	**0.5 (0.3 to 0.8)**
	Nothing	149 (31.0%)	12 (8.2%)	Reference	42 (28.6%)	Reference
Satisfaction with recovery information	Scale of 4 (least) to 20 (most)	Mean score: 13.2 (sd 3.8)	Not applicable	0.9 (0.8 to 1.0)	Not applicable	1.0 (0.9 to 1.1)
Satisfaction with support offered	Scale of 4 (least) to 20 (most)	Mean score: 12.9 (sd 3.4)	Not applicable	1.0 (0.9 to 1.2)	Not applicable	**0.9 (0.8 to 0.96)**
Expected length of clean-up	Less than a month	108 (29.9%)	1 (0.9%)	**0.1 (0.01 to 0.7)**	15 (14.2%)	0.6 (0.3 to 1.1)
	One to two months	124 (34.3%)	6 (4.8%)	0.5 (0.2 to 1.3)	27 (21.8%)	0.9 (0.5 to 1.7)
	Two months or more	129 (35.7%)	12 (9.3%)	Reference	29 (22.7%)	Reference
Impact on daily life	Some impact	68 (13.6%)	6 (8.8%)	2.0 (0.8 to 5.3)	17 (25.8%)	1.7 (0.9 to 3.1)
	No impact	432 (86.4%)	20 (4.7%)	Reference	75 (17.5%)	Reference
Trust in government	Low trust	67 (13.4%)	11 (16.7%)	**5.7 (2.5 to 13.0)**	21 (31.8%)	**2.3 (1.3 to 4.1)**
	High trust	433 (86.6%)	15 (3.5%)	Reference	71 (16.6%)	Reference

For peer review only - http://bmjopen.bmj.com/site/about/guidelines.xhtml

DRAFT – NOT FOR CIRCULATION

For peer review only

For peer review only - http://bmjopen.bmj.com/site/about/guidelines.xhtml

ICM UNLIMITED

Salisbury Telephone Survey
Final topline results
(16-04-18)

- This topline presents the findings from a survey of residents living in Salisbury, Wiltshire, conducted by ICM Unlimited.
- ICM interviewed a representative sample of 500 adults aged 18+ living in Salisbury using a telephone (CATI) methodology. The interviewing took place between 5th and 13th April 2018.
- To ensure a broadly representative sample of Salisbury residents aged 18+, quotas were set by age and gender. Data have also been weighted to the same variables.
- A sample size of 500 produces data accurate to plus or minus four (+/-4) percentage points at the 95 per cent confidence level.
- Results are based on all respondents (500) unless otherwise stated.
- An asterisk (*) denotes a value that is greater than zero but less than one.
- Where results do not sum to 100%, this may be due to multiple responses, computer rounding or the exclusion of don't knows/not stated.
- Conducted in accordance with ISO 20252 and ISO 27001, the international standards for market research and information security management.

For peer review only

For peer review only - http://bmjopen.bmj.com/site/about/guidelines.xhtml

Q2. Thinking about the events in Salisbury since 4th March 2018, how much have you seen, heard or read about what actually happened? SINGLE CODE

	%
A lot	89
A little	11
Nothing at all	*
Don't know	*

Q3. I'd like to ask you some questions about how you have been feeling over the past week in relation to the incident in Salisbury. For each of the following phrases, please tell me whether you have been feeling that way when thinking about the incident in Salisbury "Not at all, Somewhat, Moderately or Very Much". READ OUT EACH. SINGLE CODE FOR EACH OF THE 8 ITEMS BELOW

		Not at all	Somewhat	Moderately	Very much
**Calm**	%	7	14	27	52
**Tense**	%	75	12	11	3
**Upset**	%	57	17	17	9
**Relaxed**	%	10	16	33	41
**Content**	%	21	16	28	34
**Worried**	%	60	20	14	6
**Angry**	%	54	18	18	10
**Uncertain**	%	51	18	23	8

Q4. Following the incident, what is your biggest concern for the Salisbury and the community?

VERBATIMS PROVIDED

Q5. On a scale of 0 to 4, where 0 is not at all and 4 is a lot, to what degree do you feel your health is at risk as a consequence of the recent incident in Salisbury? SINGLE CODE

	%
0 - Not at all	74
1	14
2	7
3	4
4 - A lot	1
Don't know	1

For peer review only - http://bmjopen.bmj.com/site/about/guidelines.xhtml

ICM UNLIMITED

Q6. On Monday 5 March, several areas in Salisbury were cordoned off by the police as part of their investigations. These include the Mill Pub, Zizzi's restaurant, an area around a bench in The Maltings and the London Road cemetery. As far as you know, did you personally go to any of these areas between Sunday 4 March at 1:30pm and Monday 5 March at 11:30pm? MULTI CODE

	%
**Net: Yes**	**14**
Yes – General area around bench in The Maltings	8
Yes – Zizzi's restaurant	3
Yes – London Road Cemetery	2
Yes – Mill Pub	1
Yes – somewhere else	4
No	85
Not sure	1

Q7. Do you believe that you have been exposed to any of the chemical that was used in this incident? SINGLE CODE

	%
**Net: Yes**	*****
Definitely yes	0
Probably yes	*
Not sure	3
Probably no	18
Definitely no	79
**Net: No**	**96**

Q8. Do you believe that any close family members or those dear to you have been exposed to any of the chemical that was used in this incident? SINGLE CODE

	%
**Net: Yes**	**3**
Definitely yes	1
Probably yes	3
Not sure	6
Probably no	21
Definitely no	70
**Net: No**	**91**

Q9. Have you sought help or advice about the effects of the incident in Salisbury on your own health? SINGLE CODE

	%
Yes	1
No	99

For peer review only - http://bmjopen.bmj.com/site/about/guidelines.xhtml

Q10. I am going to read out a list of ways that the incident might have affected you. Please tell me if any of them apply to you. MULTICODE

	%
**Net: Have been affected**	**14**
There has been some other, serious, disruption to your day to day life	9
Your ability to go to work has been affected	4
You own a business that has been affected	2
Your income has been affected	2
None of the above	86

Q11. How much information have you seen, heard or read about the recovery support, for example the financial support package, the government has made available? SINGLE CODE

	%
A lot	18
A little	46
Nothing at all	32
Don't know	4

Q12. What measures / initiatives from the recovery support package have you seen, read or heard about?
Base: All respondents who are aware of the recovery support package (331).

VERBATIMS PROVIDED

Q13. To what extent do you believe that the information provided on the recovery support package: READ OUT EACH. SINGLE CODE FOR EACH

Base: All respondents who are aware of the recovery support package (331)

		Very clear 5	4	Neither clear nor unclear 3	2	Very unclear 1	Don't know
**A) is clear**	%	15	16	32	14	9	14
		Very timely 5	4	Neither timely nor tardy 3	2	Tardy 1	Don't know
**B) is timely**	%	16	17	38	12	9	8
		Sufficient 5	4	Neither sufficient nor insufficient 3	2	Insufficient 1	Don't know
**C) is sufficient**	%	16	12	32	10	13	17
		Very useful 5	4	Neither useful nor not useful 3	2	Not useful 1	Don't know
**D) is useful**	%	24	23	30	5	8	10

For peer review only - http://bmjopen.bmj.com/site/about/guidelines.xhtml

ICM UNLIMITED

Q14. Where would you go to get information about the recovery support package?

Base: All respondents who are aware of the recovery support package (331).

VERBATIMS PROVIDED

Q15. To what extent do you agree or disagree with the following statements. Please answer strongly agree, agree, neither agree nor disagree, disagree, or strongly disagree. READ OUT EACH. SINGLE CODE FOR EACH

		Strongly agree	Agree	Neither	Disagree	Strongly disagree	Don't know	**Net: Agree**	**Net: Disagree**
**I think that sufficient support is being offered by the government to individuals directly affected**	%	8	30	32	10	6	13	**38**	**16**
**I think that sufficient support is being offered by the government to businesses directly affected**	%	6	30	24	15	7	17	**37**	**22**
**I think that sufficient support is being offered by the government to local residents across Salisbury**	%	7	34	27	12	7	13	**41**	**19**
**I think that sufficient support is being offered by the government to local businesses across Salisbury**	%	7	32	22	18	6	16	**38**	**24**

Q16. How much information have you seen, heard or read about the clean-up / decontamination process in Salisbury? SINGLE CODE

	%
A lot	32
A little	52
Nothing at all	14
Don't know	2

For peer review only - http://bmjopen.bmj.com/site/about/guidelines.xhtml

Q17. How long would you expect the clean-up process to last? SINGLE CODE. DO NOT READ OUT OUT OPTIONS

	%
Less than a month	22
One to two months	26
Between two to six months	19
Between six months to a year	5
More than a year	1
Don't know	26

Q18. Which of these following phrases do you think best describes recent events? READ OUT. REVERSE ORDER, SINGLE CODE

	%
An incident intended to harm only a single person	54
An incident intended to harm a small number of specific people	36
An incident intended to harm the wider public	6
Don't know	4

Q19. Since the incident in Salisbury, have you…? READ OUT EACH. SINGLE CODE FOR EACH

		Yes	No	Not applicable
**Deliberately reduced the amount you go into Salisbury**	%	19	80	1
**Washed or disposed of clothing or other items, specifically because they might have been contaminated with the chemical**	%	3	95	2
**Taken any other action to protect your health, or the health of a close family member or someone dear to you**	%	5	95	1
IF YES TO 3. And what is that that you have done?	%	VERBATIMS PROVIDED		

For peer review only - http://bmjopen.bmj.com/site/about/guidelines.xhtml

ICM UNLIMITED

Q20. The next set of statements are about how government and official agencies are dealing with issues relating to the incident in Salisbury (including public safety, clean-up process and recovery support).

I'd like you to tell me whether each one is probably true, probably false or if you're not sure. READ OUT EACH. SINGLE CODE FOR EACH

		Probably true	Probably false	Not sure
**In general, I think the government / official agencies are doing a good job of dealing with the incident**	%	76	9	15
**In general, I think the government / official agencies have enough resources to cope with the incident**	%	65	17	17
**In general, I think the government / official agencies have the necessary knowledge to deal with the incident**	%	74	13	13
**In general, I think the government / official agencies are acting in the public's best interests in dealing with the incident**	%	79	10	11
**In general, I think the government / official agencies are managing the incident in a fair way**	%	75	12	13
**In general, I feel confident in the government / official agencies ability to deal with the incident**	%	73	13	14

For peer review only

For peer review only - http://bmjopen.bmj.com/site/about/guidelines.xhtml

STROBE Statement—Checklist of items that should be included in reports of ***cross-sectional studies***

	Item No	Recommendation	Page no
**Title and abstract**	1	(*a*) Indicate the study's design with a commonly used term in the title or the abstract	1
		(*b*) Provide in the abstract an informative and balanced summary of what was done and what was found	3
**Introduction**			
Background/rationale	2	Explain the scientific background and rationale for the investigation being reported	5,6
Objectives	3	State specific objectives, including any prespecified hypotheses	6
**Methods**			
Study design	4	Present key elements of study design early in the paper	7
Setting	5	Describe the setting, locations, and relevant dates, including periods of recruitment, exposure, follow-up, and data collection	7
Participants	6	(*a*) Give the eligibility criteria, and the sources and methods of selection of participants	7
Variables	7	Clearly define all outcomes, exposures, predictors, potential confounders, and effect modifiers. Give diagnostic criteria, if applicable	7-9
Data sources/ measurement	8*	For each variable of interest, give sources of data and details of methods of assessment (measurement). Describe comparability of assessment methods if there is more than one group	*7-9*
Bias	9	Describe any efforts to address potential sources of bias	7
Study size	10	Explain how the study size was arrived at	7
Quantitative variables	11	Explain how quantitative variables were handled in the analyses. If applicable, describe which groupings were chosen and why	9
Statistical methods	12	(*a*) Describe all statistical methods, including those used to control for confounding	9
		(*b*) Describe any methods used to examine subgroups and interactions	N/A
		(*c*) Explain how missing data were addressed	9
		(*d*) If applicable, describe analytical methods taking account of sampling strategy	N/A
		(*e*) Describe any sensitivity analyses	N/A
**Results**			
Participants	13*	(a) Report numbers of individuals at each stage of study—eg numbers potentially eligible, examined for eligibility, confirmed eligible, included in the study, completing follow-up, and analysed	10
		(b) Give reasons for non-participation at each stage	10
		(c) Consider use of a flow diagram	N/A
Descriptive data	14*	(a) Give characteristics of study participants (eg demographic, clinical, social) and information on exposures and potential confounders	Tables 2-5
		(b) Indicate number of participants with missing data for each variable of interest	Tables 2-5
Outcome data	15*	Report numbers of outcome events or summary measures	Tables 2-5
Main results	16	(*a*) Give unadjusted estimates and, if applicable, confounder-adjusted estimates and their precision (eg, 95% confidence interval). Make clear	Tables 2-5

For peer review only - http://bmjopen.bmj.com/site/about/guidelines.xhtml

		which confounders were adjusted for and why they were included	
		(*b*) Report category boundaries when continuous variables were categorized	Tables 2-5
		(*c*) If relevant, consider translating estimates of relative risk into absolute risk for a meaningful time period	N/A
Other analyses	17	Report other analyses done—eg analyses of subgroups and interactions, and sensitivity analyses	N/A
**Discussion**			
Key results	18	Summarise key results with reference to study objectives	12
Limitations	19	Discuss limitations of the study, taking into account sources of potential bias or imprecision. Discuss both direction and magnitude of any potential bias	13
Interpretation	20	Give a cautious overall interpretation of results considering objectives, limitations, multiplicity of analyses, results from similar studies, and other relevant evidence	12-13
Generalisability	21	Discuss the generalisability (external validity) of the study results	13
**Other information**			
Funding	22	Give the source of funding and the role of the funders for the present study and, if applicable, for the original study on which the present article is based	15

*Give information separately for exposed and unexposed groups.

Note: An Explanation and Elaboration article discusses each checklist item and gives methodological background and published examples of transparent reporting. The STROBE checklist is best used in conjunction with this article (freely available on the Web sites of PLoS Medicine at http://www.plosmedicine.org/, Annals of Internal Medicine at http://www.annals.org/, and Epidemiology at http://www.epidem.com/). Information on the STROBE Initiative is available at www.strobe-statement.org.

For peer review only

Public responses to the Salisbury Novichok incident: A cross-sectional survey of anxiety, anger, uncertainty, perceived risk and avoidance behaviour in the local community

Journal:	*BMJ Open*
Manuscript ID	bmjopen-2019-036071.R1
Article Type:	Original research
Date Submitted by the Author:	15-Jul-2020
Complete List of Authors:	Rubin, GJ; King's College London, Institute of Psychiatry, Psychology and Neuroscience<br>Webster, Rebecca; The University of Sheffield, Department of Psychology<br>Amlot, Richard; Public Health England Porton, Emergency Response Department<br>Carter, Holly; Public Health England Porton, Emergency Response Department<br>Weston, Dale; Public Health England Porton, Emergency Response Department<br>Wessely, Simon; King's College London, Institute of Psychiatry, Psychology and Neuroscience
**Primary Subject Heading**:	Public health
Secondary Subject Heading:	Public health
Keywords:	PUBLIC HEALTH, Risk management < HEALTH SERVICES ADMINISTRATION & MANAGEMENT, TOXICOLOGY

I, the Submitting Author has the right to grant and does grant on behalf of all authors of the Work (as defined in the below author licence), an exclusive licence and/or a non-exclusive licence for contributions from authors who are: i) UK Crown employees; ii) where BMJ has agreed a CC-BY licence shall apply, and/or iii) in accordance with the terms applicable for US Federal Government officers or employees acting as part of their official duties; on a worldwide, perpetual, irrevocable, royalty-free basis to BMJ Publishing Group Ltd ("BMJ") its licensees and where the relevant Journal is co-owned by BMJ to the co-owners of the Journal, to publish the Work in this journal and any other BMJ products and to exploit all rights, as set out in our licence.

The Submitting Author accepts and understands that any supply made under these terms is made by BMJ to the Submitting Author unless you are acting as an employee on behalf of your employer or a postgraduate student of an affiliated institution which is paying any applicable article publishing charge ("APC") for Open Access articles. Where the Submitting Author wishes to make the Work available on an Open Access basis (and intends to pay the relevant APC), the terms of reuse of such Open Access shall be governed by a Creative Commons licence – details of these licences and which Creative Commons licence will apply to this Work are set out in our licence referred to above.

Other than as permitted in any relevant BMJ Author's Self Archiving Policies, I confirm this Work has not been accepted for publication elsewhere, is not being considered for publication elsewhere and does not duplicate material already published. I confirm all authors consent to publication of this Work and authorise the granting of this licence.

For peer review only

Public responses to the Salisbury Novichok incident: A cross-sectional survey of anxiety, anger, uncertainty, perceived risk and avoidance behaviour in the local community

G James Rubin[1] (0000-0002-4440-0570), Rebecca K Webster[1] (0000-0002-5136-1098), Richard Amlôt[2], Holly Carter[2], Dale Weston[2], Simon Wessely[1] (0000-0002-6743-9929)

Affiliations

1: King's College London, Institute of Psychiatry, Psychology & Neuroscience, UK

2: Emergency Response Department, Public Health England, UK

Addresses

G James Rubin: King's College London, Institute of Psychiatry, Psychology and Neuroscience, Department of Psychological Medicine, Weston Education Centre, Cutcombe Road, London, SE5 9RJ, UK

Reader in the Psychology of Emerging Health Risks

Rebecca K Webster: Department of Psychology, University of Sheffield, Cathedral Court, 1 Vicar Lane, Sheffield, S1 2LT, UK

Lecturer in Psychology

Richard Amlôt: Emergency Response Department, Public Health England, Manor Farm Road, Porton Down, Salisbury, SP4 0JG

Professor of Practice in the Psychology of Health Protection

Dale Weston: Emergency Response Department, Public Health England, Manor Farm Road, Porton Down, Salisbury, SP4 0JG

Research Fellow

For peer review only - http://bmjopen.bmj.com/site/about/guidelines.xhtml

For peer review only

Holly Carter: Emergency Response Department, Public Health England, Manor Farm Road, Porton Down, Salisbury, SP4 0JG

Research Fellow

Simon Wessely: King's College London, Institute of Psychiatry, Psychology and Neuroscience, Department of Psychological Medicine, Weston Education Centre, Cutcombe Road, London, SE5 9RJ, UK

Regius Professor of Psychiatry

Corresponding author

James Rubin: gideon.rubin@kcl.ac.uk

For peer review only

For peer review only - http://bmjopen.bmj.com/site/about/guidelines.xhtml

Abstract

Objectives: Malicious incidents involving chemical agents sometimes trigger high public concern. We aimed to: 1) identify levels of emotion, perceived risk and behaviour change with regards to visiting Salisbury, one month after three people were poisoned with a nerve agent; and 2) test whether factors including receipt of information, beliefs about personal exposure and trust in government were associated with these outcomes.

Design: A cross-sectional telephone survey of a random sample of Salisbury residents.

Setting: Conducted between 5th and 13th April 2018.

Participants: 500 residents aged 18 or over.

Outcome measures: Self-reported anxiety, anger, uncertainty, perceived risk to self and avoidance of Salisbury.

Results: Any degree of anxiety, anger and uncertainty was reported by 40.6%, 29.8% and 30.6% of participants, respectively. For the majority, the level of emotion reported was mild. Only 7.0% met the criteria for high anxiety and 5.2% reported feeling any risk to their health, whereas 18.6% reported avoiding Salisbury. Factors associated with avoidance of Salisbury included being female, unable to rule out exposure for oneself, or of loved ones, believing the incident was targeted against the general public, and lower trust in the government and responding agencies. Hearing a lot or a little about the recovery support (e.g. financial packages) as opposed to nothing at all, and being satisfied with this information were associated with reduced avoidance.

Conclusions: Although the March 2018 Salisbury incident had a relatively modest impact on emotion and risk perception in the community, the number who reported avoiding the city was notable. In this, and future incidents, assuring people that contamination resulted from a targeted, rather than indiscriminate, incident; demonstrating that contamination is contained within specific areas; improving communication about any financial support; and promoting trust in responding agencies should help provide additional reassurance to the community.

For peer review only

For peer review only - http://bmjopen.bmj.com/site/about/guidelines.xhtml

Strengths and limitations of the study

- The study sample was demographically representative of adult residents living in Salisbury, UK in terms of age and gender.
- The study took place shortly after the Salisbury Novichok incident, and data collection took place over a short period of time.
- While it is possible that the sample was biased towards people with a specific interest in the incident, we attempted to reduce this by not revealing the full topic of the survey until after initial consent had been obtained.
- This study replicated previous findings about the importance of the perceived extensiveness of contamination and the perceived motivation for an incident in determining emotional and behavioural responses and risk perception.
- The response rate was extremely low, and although quota sampling may have mitigated the effects of this, whether the surveys are psychologically representative of the general population is unclear.

For peer review only

For peer review only - http://bmjopen.bmj.com/site/about/guidelines.xhtml

Introduction

Public health incidents involving chemical, biological, radiological or nuclear (CBRN) agents can sometimes trigger seemingly disproportionate levels of distress among the affected community. Previous incidents have witnessed high levels of anxiety or anger [1], spontaneous evacuations or attempts to avoid potentially contaminated areas [2 3], stigmatisation of people seen as contaminated [4 5], high levels of help-seeking among people who have not been exposed to the agent [6], and economic and social upheavals [7].

Attempts by responding agencies to reduce these effects often focus on improving communication with an affected community by identifying and addressing specific concerns (e.g.[8 9]) and by building trust with those affected (e.g. [10 11]). Research in this area has resulted in generic guidelines that can be used to assist in the acute phase of these responses, for example by identifying topics that members of the public are likely to have particular misgivings about, such as perceived exposure to a toxic substance [12 13]. Less research has focussed on the importance of other factors that may become relevant once the acute phase of a CBRN incident has passed. For example, the importance of communication about the clean-up operation or the level of public satisfaction with any financial support that is subsequently made available.

The literature on CBRN incidents is also problematic because it often relies on the use of hypothetical scenarios, rather than the study of actual incidents. For example, in a 2012 systematic review of communication research relating to the deliberate use of CBRN material [12], out of the 33 studies identified, only 12 investigated genuine incidents, 11 of which related to the US anthrax attacks of 2001. The twelfth explored the reactions of the London population to the use of radioactive Polonium 210 in the apparent assassination of the former Russian intelligence officer Alexander Litvinenko, an incident which resulted in a major public health response after a restaurant and hotel bar were found to be contaminated [9]. The study found that increased concern among members of the public was linked to the perceived motivation underlying the incident (i.e. that it had been targeted at the wider public rather than at a single individual), and to a belief that contamination might occur away from the areas that had been cordoned-off by the emergency services.

While studies on genuine incidents involving the use of CBRN agents are rare, rarer still are studies that are able to reproduce previous findings. The idiosyncrasies of each incident in terms of the population affected, public familiarity with the agent and the media narrative

For peer review only - http://bmjopen.bmj.com/site/about/guidelines.xhtml

surrounding the incident often make it difficult to compare results. But on 4 March 2018, an incident occurred in England which bore remarkable similarities to the Polonium 210 incident. Sergei Skripal, an ex-Russian intelligence officer, his daughter and a police officer became seriously ill following exposure to the nerve agent Novichok in what the UK Government described as an attempted assassination. The incident occurred in the small English city of Salisbury (population: 40,000), whose residents suddenly found themselves at the centre of a major police investigation, media furore and diplomatic crisis. Several areas of the city were cordoned off by police both to preserve forensic evidence and to prevent other members of the public from being exposed to the agent. Investigations initially focussed on a pub and restaurant that were visited by the Skripals shortly before they became ill.

A week after the cordons went up, the Chief Medical Officer for England advised that, as a precautionary measure, members of the public who had been in the pub or restaurant at the time of the incident should wash the clothes they had been wearing, wipe or wash personal items such as mobile phones or jewellery, and seal dry-clean only clothing in plastic bags. It was later announced that dry-clean only clothing would be collected and incinerated. As the clean-up operation continued, a package of financial measures was announced by the government to support individuals and businesses that had been affected by a decline in visitors into the city. It was not until 1 March 2019 that the decontamination efforts in Salisbury finally came to an end.

In this study, we used a telephone survey of the local population of Salisbury conducted one month after the incident began in order to: 1) identify levels of anxiety, anger, uncertainty, perceived risk and changes in behaviour regarding visiting Salisbury; 2) test whether factors that may be particularly relevant in the acute stage of the incident (amount heard about the incident, perceived exposure of self, perceived exposure of significant others, perceived motivation for the incident) were associated with these outcomes and 3) test whether factors related to the recovery stage of the incident were associated with these outcomes, specifically the amount heard about the clean-up or recovery effort, the satisfaction with this information or the level of support offered, the expected length of the clean-up, the level of impact on the participant's daily lives or the level of trust in the government and responding agencies.

For peer review only - http://bmjopen.bmj.com/site/about/guidelines.xhtml

Methods

Design

Between 5th and 13th April 2018, ICMUnlimited conducted a telephone survey of 500 residents living in Salisbury, UK. This sample size provided a total 95% confidence interval of approximately plus or minus 4% for each prevalence estimate. To achieve a broadly representative sample, ICMUnlimited used a random approach to ensure that all landline telephone numbers within the geographical region had an equal chance of being called, while quotas were set for the sample based on age and gender based on Census 2011 data. To reduce selection bias, participants were initially informed that the survey related to "issues affecting the UK" and were only informed after providing consent that the particular issues of interest were the recent events in Salisbury. Interviews typically lasted around 10 minutes. Calls were made during the working day, during the evenings and at the weekend to reduce selection bias associated with working outside the home.

The study was approved by King's College London's Psychiatry, Nursing and Midwifery Research Ethics Subcommittee (reference: HR-17/18-5881).

Patient and public involvement

No patient involved.

Participants

In order to take part, respondents were required to be 18 years or older, to live in Salisbury and surrounding areas (postcode areas SP1, SP2, SP3, SP4, SP5, SP6) and to speak English.

Outcomes

The supplemental material provides a copy of the wording used for all items in the survey.

Anxiety was measured using an adapted version of the six item state trait anxiety inventory (STAI-6) [14] which asked participants to report how they had been feeling over the past week "when thinking about the incident in Salisbury." The STAI-6 provides scores from 6 (least anxiety) to 24 (most anxiety). In line with previous work on public responses to major incidents [1 15], we categorised people who scored 12 or more as having anxiety about the incident and those who scored 18 or more as having high anxiety. Two additional items were added to the end of the inventory to assess anger and uncertainty. These were analysed separately, with responses of 'moderately' or 'very much' being taken as indicating the presence of anger or uncertainty.

For peer review only - http://bmjopen.bmj.com/site/about/guidelines.xhtml

Perceived risk was measured using a single item adapted from previous work on the Polonium 210 incident [9] asking participants "to what degree do you feel your health is at risk as a consequence of the recent incident in Salisbury." Responses were given on a scale of 0 (not at all) to 4 (a lot). As with the Polonium 210 incident, we categorised people who gave a response of 3 or 4 as perceiving a personal health risk.

Changes in behaviour regarding visiting Salisbury was assessed using the item: "Since the incident in Salisbury, have you deliberately reduced the amount you go into Salisbury?" Participants could respond "yes" or "no".

Predictor measures related to the acute stage of the incident

Participants were asked how much they had seen, heard or read about the incident, with response options being a lot, a little and nothing at all.

In order to assess perceived exposure, participants were asked whether they believed that they, or a close family member or someone dear to them, had been exposed to "any of the chemical that was used in this incident" (definitely yes, probably yes, not sure, probably no, definitely no).

In order to assess perceptions about the motives underlying the incident, we adapted a single item from the Polonium 210 study [9] which asked participants whether they thought the incident was intended to harm only a single person, a small number of people, or the wider public.

Predictor measures related to the recovery stage

Participants were asked how much they had seen, heard or read about the clean-up process in Salisbury and about recovery support "for example the financial support package the government has made available." For both questions, possible responses were a lot, a little and nothing at all. For those aware of the recovery support package, follow-up questions asked the extent to which they felt the information they had received was: clear; timely; sufficient; and useful. These items were summed to give a scale with acceptable internal reliability (Cronbach's alpha 0.78), from 4 (least satisfied) to 20 (most satisfied). Participants were also asked their level of agreement with statements that sufficient support was being offered by the government to: individuals directly affected, businesses directly affected, local residents across Salisbury and local business across Salisbury. Again, these items were

For peer review only - http://bmjopen.bmj.com/site/about/guidelines.xhtml

summed to give a scale with acceptable internal reliability (Cronbach's alpha 0.88) from 4 (least satisfied) to 20 (most satisfied).

Participants were asked a single open-ended item on their expectations for the length of the clean-up process.

Impact on the participant's financial or daily life was assessed by asking whether "your ability to go to work has been affected," "you own a business that has been affected," "your income has been affected," or "there has been some other, serious, disruption to your day to day life."

Trust in the official response was assessed using six items developed for use in an infectious disease outbreak [16] which assessed whether, in relation to the incident, the participant thought the government and official agencies were doing a good job, had enough resources, had the necessary knowledge, were acting in the public's best interests, were managing the incident in a fair way, and whether they felt confident about their ability to deal with the incident. Responses to these items showed acceptable reliability (Cronbach's alpha 0.74) and were summed to provide a score of 6 (least trust) to 18 (most trust), and then dichotomised into scores of low (12 or below) versus high (13 or more) trust.

Statistical analysis

For all analyses we coded responses of "don't know" or "not applicable" as missing data.

We used logistic regressions to calculate adjusted odds ratios to test the association between gender and age (controlled for each other) and each of the outcome variables, as well as for each predictor measure (controlling for gender and age) and each outcome variable.

For perceived exposure variables, we compared participants who believed they had probably or definitely been exposed or who were not sure, against those who believed they had probably or definitely not been exposed. For perceived impact, we compared those who reported impact on any aspect of their life with those who reported no impact.

Although topline data in the supplementary materials are reported weighted, tor ease of interpretation, all analyses were conducted using unweighted data.

For peer review only - http://bmjopen.bmj.com/site/about/guidelines.xhtml

Results

Response rates

In order to obtain 500 valid responses, ICMUnlimited made a total of 18,753 calls, resulting in contact with 6,808 people who were within quota (7.3%). Low response rates are typical of this style of telephone survey, and are not necessarily indicative of high response bias [17]. Two hundred and eighty participants (56.0%) were female and 220 (44.0%) male. This was roughly in line with 2011 census data for Salisbury (52% female, 48% male)[18]. One hundred and sixty-eight participants (33.6%) were aged 18 to 44, 195 (39.0%) were aged 45 to 64, and 137 (27.4%) were 65 or over. This showed an underrepresentation of 18 to 44 year olds compared to census data (43%). Seventeen participants (3%) reported having been in the pub or restaurant at the centre of incident.

Main outcomes

The main outcomes are summarised in Table 1. Two hundred and three participants (40.6%) were defined as having some level of anxiety, while anger and uncertainty were reported by 149 (29.8%) and 153 (30.6%) participants, respectively. Only 35 (7.0%) participants met the criteria for high anxiety and only 26 (5.2%) reported feeling that there was any risk to their own health. Ninety-two (18.6%) reported having deliberately reduced the amount they go into Salisbury.

Acute predictors of outcomes

Table 2 shows the associations between the various acute stage predictor variables and emotional response. In general, older adults were more likely to report anxiety and anger, while women were more likely to report anxiety. People who were unable to rule out the possibility that they themselves had been exposed to the chemical agent were more likely to report anxiety, anger and uncertainty, while people who were unable to rule out exposure amongst friends or relatives were more likely to report anxiety and uncertainty. Those who believed the incident was targeted at the wider public were more likely to report uncertainty than people who felt it was targeted at a single individual.

Although the small number of people categorised as having high anxiety prevented us from conducting detailed statistical analyses, it was notable that most people in this group had not been to a contaminated location and reported believing that neither they nor any close friends or relatives had been exposed to the nerve agent.

For peer review only - http://bmjopen.bmj.com/site/about/guidelines.xhtml

Table 3 shows the association between the acute-stage predictors and perceived risk to self or behaviour change. People who could not rule out that they themselves had been exposed to the chemical agent were more likely to feel that their health was at risk and to have avoided visiting Salisbury. The same pattern of findings was also true for those who could not rule out exposure of friends or family members, and those who felt the incident was targeted at the wider public rather than at a single person. Being female was only associated with avoidance of Salisbury, whereas participants who had heard little or no information about the incident were more likely to feel that their health was at risk.

Recovery stage predictors of outcomes

Tables 4 and 5 show the associations between recovery stage predictors and the outcomes. Greater satisfaction with the information given out about the recovery support was associated with lower levels of anxiety and uncertainty, while participants who reported having heard nothing about the recovery support or who were less satisfied with the support available had a greater likelihood of avoiding Salisbury. Those who thought the clean-up would take less than one month were more likely to perceive a lower risk to self. Lower trust in the government and official agencies was associated with a greater likelihood of anxiety, uncertainty, perceived risk to self and avoiding Salisbury.

For peer review only

Discussion

One month following the Novichok incident in Salisbury, many members of the local community reported some degree of anxiety (40.6%), anger (29.8%) or uncertainty (30.6%). By way of comparison, using the same scale, 23.7% of the British population reported some anxiety at the very earliest stages of the swine flu outbreak [15]. The number reporting high anxiety was relatively low, however, at 7.0%. Again, for comparison, the number reporting high anxiety among the general public of Great Britain during the early stages of the swine flu outbreak was 2.1% [15], while amongst British nationals in Japan at the time of the Fukushima nuclear disaster it was 29.7% [1]. Levels of perceived risk were also low (5.2%) and slightly lower than those found during the Polonium 210 incident (11.7% [9]). Despite emotional responses being relatively low however, behavioural change was apparent, with 18.6% of people from the local area reporting deliberately reducing the amount they went into Salisbury. This is similar to the 20% of Londoners who reported intending to going into central London less often after the 7/7 bombings [19].

Our analyses suggested that there were three core issues underlying these findings. First, a pattern of associations was identified between the outcomes and believing that exposure might have occurred to oneself or a loved one, or feeling that the incident had been targeted at the wider public. Tellingly, most people reporting high anxiety had not been present within an area that was later cordoned off. Similar findings were also observed during the Polonium 210 incident [9] and suggest a level of concern that contamination may deliberately or accidentally occur beyond the areas identified by the responding agencies. Emphasising that this is unlikely should reduce levels of concern among the population, but responding agencies may need to exercise caution about this. In this incident, on 3 July 2018, two further members of the public became ill following accidental exposure to a source of Novichok from a location outside of the initial cordons. One subsequently died. As a result, multiple new locations were cordoned off by police, and members of the public were once again issued with advice about washing clothes and possessions.

The second core issue underlying concern related to the level of support being provided to the community during the recovery phase. The incident had implications for the economic wellbeing of the community, with local businesses and their employees affected by the presence of police cordons and a reduction in the number of visitors to the city. Although several measures were introduced to offset this, at the time of our survey most people had

For peer review only - http://bmjopen.bmj.com/site/about/guidelines.xhtml

heard little or nothing about them. This in turn appeared to underlie some of the anxiety and uncertainty reported by residents. Interestingly, an association was also found between having heard more about the recovery package or satisfaction with the support available, and being less likely to avoid Salisbury. Whether the information provided reduced concerns about visiting the city, or whether people who visited Salisbury were more likely to encounter information about the recovery effort is unclear. Nonetheless, ensuring that communication about recovery support is widely disseminated should be beneficial in future incidents.

The third core issue related to trust. Strong associations were observed between greater trust in the government and responding agencies' response to the incident and levels of anxiety, perceived risk and behaviour change. The importance of trust in determining public responses to public health crises has been noted in several previous studies [11 15 20 21]. Trust is not necessarily a unitary construct and appears to be influenced by multiple factors [21]. In our study we assessed several components, including perceived competence, expertise, benevolence, equity, and access to sufficient resources. All of these showed room for improvement. Demonstrating that the official agencies can be trusted across all of these factors is likely to reduce public concern.

Several limitations should be borne in mind when considering our findings. In particular, whether the sample was representative of the population surrounding Salisbury is unknown. Although the response rate was low, the use of quotas for age and gender is likely to have mitigated selection bias and while it is possible that the sample was biased towards people with a specific interest in the incident, we attempted to reduce this by not revealing the full topic of the survey until after initial consent had been obtained. Nonetheless, the underrepresentation of people aged 18 to 44 in comparison to older age groups was notable. This may relate to a lower proportion within this group who own a landline telephone. Given that older age was associated with anxiety and anger, it could be that this bias has led us to over-estimate the psychological impact of the incident. The number of analyses we conducted is a second limitation, raising the possibility of a Type I error. Caution is required in interpreting some of our findings involving confidence intervals that approached one. A third limitation relates to the fact that the survey interviewed a sample from the general population of Salisbury and surrounding areas, and did not focus specifically on those most affected by the incident. Care should be taken not to extrapolate our findings relating to the wider community to specific subsections within it, who may, for example, have had a qualitatively

For peer review only - http://bmjopen.bmj.com/site/about/guidelines.xhtml

different set of concerns and who may have received more detailed information and support from official agencies.

Overall, the initial Salisbury incident appeared to have had a relatively modest impact on levels of emotion, and risk perception, although the number who reported avoiding the city was notable. Emotional and behavioural responses were related to perceptions around the possible spread of contamination, the motives underlying the incident, the amount and quality of information received about the recovery and support efforts and levels of trust in government. As the residents of Salisbury have rightly attested, such responses should not be misconstrued as "panic."[22] In future incidents, demonstrating that contamination is known to be contained within specific areas, improving communication about any financial recovery package and promoting trust in responding agencies should all help in providing additional reassurance to the community.

For peer review only

For peer review only - http://bmjopen.bmj.com/site/about/guidelines.xhtml

Acknowledgements

We would like to thank the study participants for giving up their time to support this study, and Joe Thomson from the Research Information and Communications Unit of the Home Office for his support with this work.

Author contributions

GJR, RA, HC, DW and SW contributed equally to conceptualisation and design of the study. ICMUnlimited were responsible for the acquisition of the data. GJR and RKW conducted the data analysis and interpretation of the data. GJR wrote the first draft of the manuscript with input from RKW, RA, HC, DW and SW. GJR, RKW, RA, HC, DW and SW all approved the final version of the manuscript. GJR accepts full responsibility for the work and conduct of the study, had access to the data, and controlled the decision to publish. The corresponding author attests that all listed authors meet authorship criteria and that no others meeting the criteria have been omitted.

Role of study sponsor and funder

The sponsor and funder of the study had no role in study design, data collection, data analysis, data interpretation, or writing of the report. The corresponding author had full access to all the data in the study and had final responsibility for the decision to submit for publication.

Declaration of interests

Rubin has received a payment from the UK Government to provide advice about public responses to the Salisbury incident.

Financial disclosure statements

The research was part funded by the National Institute for Health Research Health Protection Research Units (NIHR HPRU) in Emergency Preparedness and Response (award number: HPRU-2012-10414) at King's College London, and Modelling Methodology (award number: HPRU-2012-10080) at Imperial College London, in partnership with Public Health England (PHE), and part funded by DEFRA.

The views expressed are those of the author(s) and not necessarily those of the NHS, the NIHR, the Department of Health, DEFRA or Public Health England.

For peer review only - http://bmjopen.bmj.com/site/about/guidelines.xhtml

Copyright statement

The Corresponding Author has the right to grant on behalf of all authors and does grant on behalf of all authors, a worldwide licence to the Publishers and its licensees in perpetuity, in all forms, formats and media (whether known now or created in the future), to i) publish, reproduce, distribute, display and store the Contribution, ii) translate the Contribution into other languages, create adaptations, reprints, include within collections and create summaries, extracts and/or, abstracts of the Contribution, iii) create any other derivative work(s) based on the Contribution, iv) to exploit all subsidiary rights in the Contribution, v) the inclusion of electronic links from the Contribution to third party material where-ever it may be located; and, vi) licence any third party to do any or all of the above

Declaration of competing interests

All authors have completed the ICMJE uniform disclosure form at www.icmje.org/coi_disclosure.pdf and declare: all authors had financial support from the NIHR HPRU for the submitted work; no financial relationships with any organisations that might have an interest in the submitted work in the previous three years; no other relationships or activities that could appear to have influenced the submitted work.

Full access to data

All authors had full access to all of the data in the study and can take responsibility for the integrity of the data and the accuracy of the data analysis.

Transparency declaration

The lead author confirms that this manuscript is an honest, accurate and transparent account of the study being reported. No important aspects of the study have been omitted and any discrepancies from the study as planned have been explained.

Data sharing

Data are available on reasonable request.

For peer review only

References

1. Rubin GJ, Amlôt R, Wessely S, et al. Anxiety, distress and anger among British nationals in Japan following the Fukushima nuclear accident. *Br J Psychiatry* 2012;201(5):400-07. doi: 10.1192/bjp.bp.112.111575
2. Rubin GJ, Dickmann P. How to reduce the impact of "low-risk patients" following a bioterrorist incident: Lessons from SARS, anthrax, and pneumonic plague. *Biosecur Bioterrorism* 2010;8(1):37-43. doi: 10.1089/bsp.2009.0059
3. Ziegler DJ, Brunn SD, Johnson JH. Evacuation from a technological disaster. *Geogr Rev* 1981;71(1):1-16.
4. Person B, Sy F, Holton K, et al. Fear and stigma: The epidemic within the SARS outbreak. *Emerg Infect Dis* 2004;10(2):358-63.
5. Bromet EJ. Emotional consequences of nuclear power plant disasters. *Health Phys* 2014;106(2):206-10. doi: http://dx.doi.org/10.1097/HP.0000000000000012
6. Engel CC, Locke S, Reissman DB, et al. Terrorism, trauma and mass casualty triage: How might we solve the latest mind-body problem? *Biosecur Bioterrorism* 2007;5(2):155-63.
7. Collins DL, de Carvalho AB. Chronic stress from the Goiania 137Cs radiation accident. *Behav Med* 1993;18:149-57.
8. Rubin GJ, Potts HWW, Michie S. The impact of communications about swine flu (influenza A HINIv) on public responses to the outbreak: Results from 36 national telephone surveys in the UK. *Health Technol Assess* 2010;14(34):183-266. doi: 10.3310/hta14340-03
9. Rubin GJ, Page L, Morgan O, et al. Public information needs after the poisoning of Alexander Litvinenko with polonium-210 in London: Cross sectional telephone survey and qualitative analysis. *Br Med J* 2007;335(7630):1143-46. doi: 10.1136/bmj.39367.455243.BE
10. Peters RG. A study of the factors determining perceptions of trust and credibility in environmental risk communication: the importance of overcoming negative stereotypes. *Int Arch Occup Environ Health* 1996;68:442-47.
11. Meredith LS, Eisenman DP, Rhodes H, et al. Trust influences response to public health messages during a bioterrorist event. *J Health Commun* 2007;12:217-32.
12. Rubin GJ, Chowdhury AK, Amlôt R. How to communicate with the public about chemical, biological, radiological, or nuclear terrorism: A systematic review of the literature. *Biosecur Bioterrorism* 2012;10(4):383-95. doi: 10.1089/bsp.2012.0043
13. Hyer RH, Covello VT. Effective media communication during public health emergencies. Geneva: World Health Organisation 2005.
14. Marteau TM, Becker H. The development of a six-item short-form of the state scale of the Speilberger state-trait anxiety inventory (STAI). *Br J Clin Psychol* 1992;31:301-06.
15. Rubin GJ, Amlot R, Page L, et al. Public perceptions, anxiety, and behaviour change in relation to the swine flu outbreak: cross sectional telephone survey. *Br Med J* 2009;339:b2651. doi: http://dx.doi.org/10.1136/bmj.b2651
16. Rubin GJ, Bakhshi S, Amlot R, et al. The design of a survey questionnaire to measure perceptions and behaviour during an influenza pandemic: the Flu TElephone Survey Template (FluTEST). Southampton UK: Queen's Printer and Controller of HMSO 2014.
17. O'Cathain A, Knowles E, Nicholl J. Testing survey methodology to measure patients' experiences and views of the emergency and urgent care system: Telephone versus postal survey. *BMC Med Res Methodol* 2010;10(52)

For peer review only - http://bmjopen.bmj.com/site/about/guidelines.xhtml

18. Wiltshire Census 2011 Selected Statistics Profile Tool: Salisbury Community Area. Wiltshire Council. May 2013.
19. Rubin GJ, Brewin CR, Greenberg N, et al. Psychological and behavioural reactions to the bombings in London on 7 July 2005: cross sectional survey of a representative sample of Londoners. *BMJ* 2005;331(7517):606. doi: 10.1136/bmj.38583.728484.3A
20. Tang CSK, Wong C-Y. An outbreak of the severe acute respiratory syndrome: predictors of health behaviors and effect of community prevention measures in Hong Kong, China. *Am J Public Health* 2003;93(11):1887-88.
21. Wray R, Rivers J, Whitworth A, et al. Public perceptions about trust in emergency risk communication: Qualitative research findings. *Int J Mass Emerg Disasters* 2006;24(1):45-75.
22. Morris S, Bannock C. 'The snow caused more panic': Salisbury shrugs off attack. *The Guardian* 2018.

For peer review only

For peer review only - http://bmjopen.bmj.com/site/about/guidelines.xhtml

Table 1: Emotional and behavioural reactions to the Salisbury poisoning

Emotional or behavioural outcome	Number reporting that outcome / number with valid response (%)
High or moderate anxiety	203 / 500 (40.6%)
High anxiety	35 / 500 (7.0%)
Anger	149 / 500 (29.8%)
Uncertainty	153 / 500 (30.6%)
Perceived personal risk	26 / 498 (5.2%)
Reduced amount you go into Salisbury	92 / 494 (18.6%)

For peer review only

For peer review only - http://bmjopen.bmj.com/site/about/guidelines.xhtml

Table 2: The association between acute stage predictor measures and emotional outcomes (odds ratios are adjusted for age and gender)

Variable	Variable levels	No (%) of participants	No (%) reporting anxiety	Odds ratio (95% confidence interval)	No (%) reporting anger	Odds ratio (95% confidence interval)	No (%) reporting uncertainty	Odds ratio (95% confidence interval)
Age	65+	137 (27.4%)	72 (52.6%)	**2.2 (1.4 to 3.5)**	65 (40.9%)	**2.6 (1.5 to 4.2)**	46 (33.6%)	1.5 (0.9 to 2.5)
	45-64	195 (39.0%)	72 (36.9%)	1.1 (0.7 to 1.7)	57 (29.2%)	1.5 (0.9 to 2.4)	64 (32.8%)	1.4 (0.9 to 2.2)
	18-44	168 (33.6%)	59 (35.1%)	Reference	36 (21.4%)	Reference	43 (25.6%)	Reference
Gender	Female	280 (56.0%)	129 (46.1%)	**1.8 (1.3 to 2.6)**	84 (30.0%)	1.1 (0.7 to 1.6)	89 (31.8%)	1.2 (0.8 to 17)
	Male	220 (44.0%)	74 (33.6%)	Reference	65 (29.5%)	Reference	64 (29.1%)	Reference
How much heard	A lot	449 (90.2%)	180 (40.1%)	0.8 (0.5 to 1.5)	129 (28.7%)	0.7 (0.3 to 1.2)	140 (31.2%)	1.6 (0.8 to 3.2)
	A little / nothing	49 (9.8%)	23 (46.9%)	Reference	19 (38.8%)	Reference	11 (22.4%)	Reference
Belief in exposure (self)	Definitely, probably or not sure	21 (4.2%)	16 (76.2%)	**5.5 (2.0 to 15.7)**	11 (52.4%)	**2.9 (1.2 to 7.0)**	14 (66.7%)	**4.9 (1.9 to 12.4)**
	Definitely or probably not	479 (95.8%)	187 (39.0%)	Reference	138 (28.8%)	Reference	139 (29.0%)	Reference
Belief in exposure (others)	Definitely, probably or not sure	44 (8.8%)	27 (61.4%)	**2.6 (1.3 to 4.9)**	18 (40.9%)	1.8 (0.96 to 3.5)	26 (59.1%)	**3.8 (2.0 to 7.2)**
	Definitely or probably not	456 (91.2%)	176 (38.6%)	Reference	131 (28.7%)	Reference	127 (27.9%)	Reference
Incident intended to harm…	Wider public	31 (6.2%)	18 (58.1%)	2.1 (0.96 to 4.5)	13 (41.9%)	1.5 (0.7 to 3.2)	16 (51.6%)	**2.5 (1.2 to 5.3)**
	Small number of specific people	187 (37.4%)	80 (42.8%)	1.2 (0.8 to 1.8)	53 (28.3%)	0.9 (0.6 to 1.4)	56 (29.9%)	1.0 (0.7 to 1.5)
	A single person	265 (54.9%)	101 (38.1%)	Reference	81 (30.6%)	Reference	78 (29.4%)	Reference

For peer review only - http://bmjopen.bmj.com/site/about/guidelines.xhtml

Table 3: The association between acute stage predictor measures, risk perception and behaviour (odds ratios are adjusted for age and gender

Variable	Variable levels	No (%) of participants	No (%) reporting personal risk perception	Odds ratio (95% confidence interval)	No (%) reporting avoiding Salisbury	Odds ratio (95% confidence interval)
Age	65+	137 (27.4%)	9 (6.6%)	1.9 (0.7 to 5.6)	20 (15.2%)	0.7 (0.4 to 1.2)
	45-64	195 (39.0%)	11 (5.7%)	1.6 (0.6 to 4.4)	35 (17.9%)	0.8 (0.5 to 1.3)
	18-44	168 (33.6%)	6 (3.6%)	Reference	37 (22.2%)	Reference
Gender	Female	280 (56.0%)	16 (5.7%)	1.3 (0.6 to 3.0)	61 (22.1%)	**1.7 (1.1 to 2.7)**
	Male	220 (44.0%)	10 (4.6%)	Reference	31 (14.2%)	Reference
How much heard	A lot	449 (90.2%)	20 (4.5%)	**0.3 (0.1 to 0.9)**	84 (18.8%)	1.2 (0.5 to 2.7)
	A little / nothing	49 (9.8%)	6 (12.2%)	Reference	8 (17.4%)	Reference
Belief in exposure (self)	Definitely, probably or not sure	21 (4.2%)	6 (28.6%)	**9.3 (3.2 to 26.9)**	9 (42.9%)	**3.5 (1.4 to 8.8)**
	Definitely or probably not	479 (95.8%)	20 (4.2%)	Reference	83 (17.5%)	Reference
Belief in exposure (others)	Definitely, probably or not sure	44 (8.8%)	7 (15.9%)	**4.4 (1.7 to 11.4)**	19 (45.2%)	**4.0 (2.0 to 7.7)**
	Definitely or probably not	456 (91.2%)	19 (4.2%)	Reference	73 (16.2%)	Reference
Incident intended to harm…	Wider public	31 (6.2%)	7 (22.6%)	**7.8 (2.6 to 22.9)**	13 (44.8%)	**5.0 (2.2 to 11.3)**
	Small number of specific people	187 (37.4%)	8 (4.3%)	1.2 (0.5 to 3.4)	37 (20.2%)	1.4 (0.9 to 2.3)
	A single person	265 (54.9%)	9 (3.4%)	Reference	40 (15.1%)	Reference

For peer review only - http://bmjopen.bmj.com/site/about/guidelines.xhtml

For peer review only

Table 4: The association between recovery predictor measures and emotional outcomes (odds ratios are adjusted for age and gender)

Variable	Variable levels	No (%) of participants	No (%) reporting anxiety	Odds ratio (95% confidence interval)	No (%) reporting anger	Odds ratio (95% confidence interval)	No (%) reporting uncertainty	Odds ratio (95% confidence interval)
How much heard - clean-up	A lot	169 (34.3%)	75 (44.4%)	0.9 (0.5 to 1.5)	56 (33.1%)	1.3 (0.7 to 2.5)	56 (33.1%)	1.3 (0.7 to 2.6)
	A little	257 (52.2%)	95 (37.0%)	0.7 (0.4 to 1.2)	73 (28.4%)	1.2 (0.6 to 2.2)	78 (30.4%)	1.2 (0.7 to 2.3)
	Nothing	66 (13.4%)	30 (45.5%)	Reference	17 (25.8%)	Reference	17 (25.8%)	Reference
How much heard - recovery	A lot	96 (20.0%)	35 (36.5%)	**0.5 (0.3 to 0.9)**	30 (31.3%)	0.9 (0.5 to 1.5)	25 (26.0%)	0.6 (0.3 to 1.04)
	A little	235 (49.0%)	93 (39.6%)	0.7 (0.4 to 1.03)	70 (29.8%)	0.9 (0.5 to 1.4)	72 (30.6%)	0.8 (0.5 to 1.2)
	Nothing	149 (31.0%)	69 (46.3%)	Reference	45 (30.2%)	Reference	52 (34.9%)	Reference
Satisfaction with recovery information	Scale of 4 (least) to 20 (most)	Mean score: 13.2 (sd 3.8)	Not applicable	1.0 (0.9 to 1.04)	Not applicable	0.9 (0.9 to 1.002)	Not applicable	1.0 (0.9 to 1.07)
Satisfaction with support offered	Scale of 4 (least) to 20 (most)	Mean score: 12.9 (sd 3.4)	Not applicable	**0.9 (0.9 to 0.99)**	Not applicable	1.0 (0.9 to 1.02)	Not applicable	**0.9 (0.9 to 0.98)**
Expected length of clean-up	Less than a month	108 (29.9%)	39 (36.1%)	0.8 (0.5 to 1.4)	28 (25.9%)	0.9 (0.5 to 1.5)	40 (37.0%)	1.6 (0.9 to 2.8)
	One to two months	124 (34.3%)	50 (40.3%)	1.0 (0.6 to 1.6)	40 (32.3%)	1.2 (0.7 to 2.1)	39 (31.5%)	1.3 (0.8 to 2.2)
	Two months or more	129 (35.7%)	52 (40.3%)	Reference	37 (28.7%)	Reference	34 (26.4%)	Reference
Impact on daily life	Some impact	68 (13.6%)	33 (48.5%)	1.5 (0.9 to 2.6)	23 (33.8%)	1.3 (0.7 to 2.2)	25 (36.8%)	1.4 (0.8 to 2.4)
	No impact	432 (86.4%)	170 (39.4%)	Reference	126 (29.2%)	Reference	128 (29.6%)	Reference
Trust in government	Low trust	67 (13.4%)	35 (52.2%)	**1.8 (1.04 to 3.0)**	23 (34.3%)	1.3 (0.8 to 2.3)	24 (35.8%)	1.3 (0.8 to 2.3)
	High trust	433 (86.6%)	168 (38.8%)	Reference	126 (29.1%)	Reference	129 (29.8%)	Reference

For peer review only - http://bmjopen.bmj.com/site/about/guidelines.xhtml

Table 5: The association between recovery predictor measures, risk perception and behaviour (odds ratios are adjusted for age and gender

Variable	Variable levels	No (%) of participants	No (%) reporting personal risk perception	Odds ratio (95% confidence interval)	No (%) reporting avoiding Salisbury	Odds ratio (95% confidence interval)
How much heard - clean-up	A lot	169 (34.3%)	9 (5.3%)	1.1 (0.3 to 4.1)	32 (19.2%)	0.8 (0.4 to 1.6)
	A little	257 (52.2%)	13 (5.1%)	1.1 (0.3 to 3.9)	45 (17.6%)	0.7 (0.4 to 1.4)
	Nothing	66 (13.4%)	3 (4.6%)	Reference	15 (23.1%)	Reference
How much heard - recovery	A lot	96 (20.0%)	3 (3.1%)	0.3 (0.1 to 1.1)	12 (12.5%)	**0.4 (0.2 to 0.8)**
	A little	235 (49.0%)	11 (4.7%)	0.5 (0.2 to 1.1)	35 (15.2%)	**0.5 (0.3 to 0.8)**
	Nothing	149 (31.0%)	12 (8.2%)	Reference	42 (28.6%)	Reference
Satisfaction with recovery information	Scale of 4 (least) to 20 (most)	Mean score: 13.2 (sd 3.8)	Not applicable	0.9 (0.8 to 1.0)	Not applicable	1.0 (0.9 to 1.1)
Satisfaction with support offered	Scale of 4 (least) to 20 (most)	Mean score: 12.9 (sd 3.4)	Not applicable	1.0 (0.9 to 1.2)	Not applicable	**0.9 (0.8 to 0.96)**
Expected length of clean-up	Less than a month	108 (29.9%)	1 (0.9%)	**0.1 (0.01 to 0.7)**	15 (14.2%)	0.6 (0.3 to 1.1)
	One to two months	124 (34.3%)	6 (4.8%)	0.5 (0.2 to 1.3)	27 (21.8%)	0.9 (0.5 to 1.7)
	Two months or more	129 (35.7%)	12 (9.3%)	Reference	29 (22.7%)	Reference
Impact on daily life	Some impact	68 (13.6%)	6 (8.8%)	2.0 (0.8 to 5.3)	17 (25.8%)	1.7 (0.9 to 3.1)
	No impact	432 (86.4%)	20 (4.7%)	Reference	75 (17.5%)	Reference
Trust in government	Low trust	67 (13.4%)	11 (16.7%)	**5.7 (2.5 to 13.0)**	21 (31.8%)	**2.3 (1.3 to 4.1)**
	High trust	433 (86.6%)	15 (3.5%)	Reference	71 (16.6%)	Reference

For peer review only

For peer review only

For peer review only - http://bmjopen.bmj.com/site/about/guidelines.xhtml

Salisbury Telephone Survey
Final topline results
(16-04-18)

- This topline presents the findings from a survey of residents living in Salisbury, Wiltshire, conducted by ICM Unlimited.
- ICM interviewed a representative sample of 500 adults aged 18+ living in Salisbury using a telephone (CATI) methodology. The interviewing took place between 5th and 13th April 2018.
- To ensure a broadly representative sample of Salisbury residents aged 18+, quotas were set by age and gender. Data have also been weighted to the same variables.
- A sample size of 500 produces data accurate to plus or minus four (+/-4) percentage points at the 95 per cent confidence level.
- Results are based on all respondents (500) unless otherwise stated.
- An asterisk (*) denotes a value that is greater than zero but less than one.
- Where results do not sum to 100%, this may be due to multiple responses, computer rounding or the exclusion of don't knows/not stated.
- Conducted in accordance with ISO 20252 and ISO 27001, the international standards for market research and information security management.

For peer review only

For peer review only - http://bmjopen.bmj.com/site/about/guidelines.xhtml

Q2. Thinking about the events in Salisbury since 4th March 2018, how much have you seen, heard or read about what actually happened? SINGLE CODE

	%
A lot	89
A little	11
Nothing at all	*
Don't know	*

Q3. I'd like to ask you some questions about how you have been feeling over the past week in relation to the incident in Salisbury. For each of the following phrases, please tell me whether you have been feeling that way when thinking about the incident in Salisbury "Not at all, Somewhat, Moderately or Very Much". READ OUT EACH. SINGLE CODE FOR EACH OF THE 8 ITEMS BELOW

		Not at all	Somewhat	Moderately	Very much
**Calm**	%	7	14	27	52
**Tense**	%	75	12	11	3
**Upset**	%	57	17	17	9
**Relaxed**	%	10	16	33	41
**Content**	%	21	16	28	34
**Worried**	%	60	20	14	6
**Angry**	%	54	18	18	10
**Uncertain**	%	51	18	23	8

Q4. Following the incident, what is your biggest concern for the Salisbury and the community?

VERBATIMS PROVIDED

Q5. On a scale of 0 to 4, where 0 is not at all and 4 is a lot, to what degree do you feel your health is at risk as a consequence of the recent incident in Salisbury? SINGLE CODE

	%
0 - Not at all	74
1	14
2	7
3	4
4 - A lot	1
Don't know	1

For peer review only - http://bmjopen.bmj.com/site/about/guidelines.xhtml

ICM UNLIMITED

Q6. On Monday 5 March, several areas in Salisbury were cordoned off by the police as part of their investigations. These include the Mill Pub, Zizzi's restaurant, an area around a bench in The Maltings and the London Road cemetery. As far as you know, did you personally go to any of these areas between Sunday 4 March at 1:30pm and Monday 5 March at 11:30pm? MULTI CODE

	%
**Net: Yes**	**14**
Yes – General area around bench in The Maltings	8
Yes – Zizzi's restaurant	3
Yes – London Road Cemetery	2
Yes – Mill Pub	1
Yes – somewhere else	4
No	85
Not sure	1

Q7. Do you believe that you have been exposed to any of the chemical that was used in this incident? SINGLE CODE

	%
**Net: Yes**	*****
Definitely yes	0
Probably yes	*
Not sure	3
Probably no	18
Definitely no	79
**Net: No**	**96**

Q8. Do you believe that any close family members or those dear to you have been exposed to any of the chemical that was used in this incident? SINGLE CODE

	%
**Net: Yes**	**3**
Definitely yes	1
Probably yes	3
Not sure	6
Probably no	21
Definitely no	70
**Net: No**	**91**

Q9. Have you sought help or advice about the effects of the incident in Salisbury on your own health? SINGLE CODE

	%
Yes	1
No	99

For peer review only - http://bmjopen.bmj.com/site/about/guidelines.xhtml

ICM UNLIMITED

Q10. I am going to read out a list of ways that the incident might have affected you. Please tell me if any of them apply to you. MULTICODE

	%
**Net: Have been affected**	**14**
There has been some other, serious, disruption to your day to day life	9
Your ability to go to work has been affected	4
You own a business that has been affected	2
Your income has been affected	2
None of the above	86

Q11. How much information have you seen, heard or read about the recovery support, for example the financial support package, the government has made available? SINGLE CODE

	%
A lot	18
A little	46
Nothing at all	32
Don't know	4

Q12. What measures / initiatives from the recovery support package have you seen, read or heard about?
Base: All respondents who are aware of the recovery support package (331).

VERBATIMS PROVIDED

Q13. To what extent do you believe that the information provided on the recovery support package: READ OUT EACH. SINGLE CODE FOR EACH

Base: All respondents who are aware of the recovery support package (331)

		Very clear 5	4	Neither clear nor unclear 3	2	Very unclear 1	Don't know
**A) is clear**	%	15	16	32	14	9	14
		Very timely 5	4	Neither timely nor tardy 3	2	Tardy 1	Don't know
**B) is timely**	%	16	17	38	12	9	8
		Sufficient 5	4	Neither sufficient nor insufficient 3	2	Insufficient 1	Don't know
**C) is sufficient**	%	16	12	32	10	13	17
		Very useful 5	4	Neither useful nor not useful 3	2	Not useful 1	Don't know
**D) is useful**	%	24	23	30	5	8	10

For peer review only - http://bmjopen.bmj.com/site/about/guidelines.xhtml

ICM UNLIMITED

Q14. Where would you go to get information about the recovery support package?

Base: All respondents who are aware of the recovery support package (331).

VERBATIMS PROVIDED

Q15. To what extent do you agree or disagree with the following statements. Please answer strongly agree, agree, neither agree nor disagree, disagree, or strongly disagree. READ OUT EACH. SINGLE CODE FOR EACH

		Strongly agree	Agree	Neither	Disagree	Strongly disagree	Don't know	**Net: Agree**	**Net: Disagree**
**I think that sufficient support is being offered by the government to individuals directly affected**	%	8	30	32	10	6	13	**38**	**16**
**I think that sufficient support is being offered by the government to businesses directly affected**	%	6	30	24	15	7	17	**37**	**22**
**I think that sufficient support is being offered by the government to local residents across Salisbury**	%	7	34	27	12	7	13	**41**	**19**
**I think that sufficient support is being offered by the government to local businesses across Salisbury**	%	7	32	22	18	6	16	**38**	**24**

Q16. How much information have you seen, heard or read about the clean-up / decontamination process in Salisbury? SINGLE CODE

	%
A lot	32
A little	52
Nothing at all	14
Don't know	2

For peer review only - http://bmjopen.bmj.com/site/about/guidelines.xhtml

ICM UNLIMITED

Q17. How long would you expect the clean-up process to last? SINGLE CODE. DO NOT READ OUT OUT OPTIONS

	%
Less than a month	22
One to two months	26
Between two to six months	19
Between six months to a year	5
More than a year	1
Don't know	26

Q18. Which of these following phrases do you think best describes recent events? READ OUT. REVERSE ORDER, SINGLE CODE

	%
An incident intended to harm only a single person	54
An incident intended to harm a small number of specific people	36
An incident intended to harm the wider public	6
Don't know	4

Q19. Since the incident in Salisbury, have you…? READ OUT EACH. SINGLE CODE FOR EACH

		Yes	No	Not applicable
**Deliberately reduced the amount you go into Salisbury**	%	19	80	1
**Washed or disposed of clothing or other items, specifically because they might have been contaminated with the chemical**	%	3	95	2
**Taken any other action to protect your health, or the health of a close family member or someone dear to you**	%	5	95	1
IF YES TO 3. And what is that that you have done?	%	VERBATIMS PROVIDED		

For peer review only - http://bmjopen.bmj.com/site/about/guidelines.xhtml

ICM UNLIMITED

Q20. The next set of statements are about how government and official agencies are dealing with issues relating to the incident in Salisbury (including public safety, clean-up process and recovery support).

I'd like you to tell me whether each one is probably true, probably false or if you're not sure. READ OUT EACH. SINGLE CODE FOR EACH

		Probably true	Probably false	Not sure
**In general, I think the government / official agencies are doing a good job of dealing with the incident**	%	76	9	15
**In general, I think the government / official agencies have enough resources to cope with the incident**	%	65	17	17
**In general, I think the government / official agencies have the necessary knowledge to deal with the incident**	%	74	13	13
**In general, I think the government / official agencies are acting in the public's best interests in dealing with the incident**	%	79	10	11
**In general, I think the government / official agencies are managing the incident in a fair way**	%	75	12	13
**In general, I feel confident in the government / official agencies ability to deal with the incident**	%	73	13	14

For peer review only

For peer review only - http://bmjopen.bmj.com/site/about/guidelines.xhtml

STROBE Statement—Checklist of items that should be included in reports of ***cross-sectional studies***

	Item No	Recommendation	Page no
**Title and abstract**	1	(*a*) Indicate the study's design with a commonly used term in the title or the abstract	1
		(*b*) Provide in the abstract an informative and balanced summary of what was done and what was found	3
**Introduction**			
Background/rationale	2	Explain the scientific background and rationale for the investigation being reported	5,6
Objectives	3	State specific objectives, including any prespecified hypotheses	6
**Methods**			
Study design	4	Present key elements of study design early in the paper	7
Setting	5	Describe the setting, locations, and relevant dates, including periods of recruitment, exposure, follow-up, and data collection	7
Participants	6	(*a*) Give the eligibility criteria, and the sources and methods of selection of participants	7
Variables	7	Clearly define all outcomes, exposures, predictors, potential confounders, and effect modifiers. Give diagnostic criteria, if applicable	7-9
Data sources/ measurement	8*	For each variable of interest, give sources of data and details of methods of assessment (measurement). Describe comparability of assessment methods if there is more than one group	*7-9*
Bias	9	Describe any efforts to address potential sources of bias	7
Study size	10	Explain how the study size was arrived at	7
Quantitative variables	11	Explain how quantitative variables were handled in the analyses. If applicable, describe which groupings were chosen and why	9
Statistical methods	12	(*a*) Describe all statistical methods, including those used to control for confounding	9
		(*b*) Describe any methods used to examine subgroups and interactions	N/A
		(*c*) Explain how missing data were addressed	9
		(*d*) If applicable, describe analytical methods taking account of sampling strategy	N/A
		(*e*) Describe any sensitivity analyses	N/A
**Results**			
Participants	13*	(a) Report numbers of individuals at each stage of study—eg numbers potentially eligible, examined for eligibility, confirmed eligible, included in the study, completing follow-up, and analysed	10
		(b) Give reasons for non-participation at each stage	10
		(c) Consider use of a flow diagram	N/A
Descriptive data	14*	(a) Give characteristics of study participants (eg demographic, clinical, social) and information on exposures and potential confounders	Tables 2-5
		(b) Indicate number of participants with missing data for each variable of interest	Tables 2-5
Outcome data	15*	Report numbers of outcome events or summary measures	Tables 2-5
Main results	16	(*a*) Give unadjusted estimates and, if applicable, confounder-adjusted estimates and their precision (eg, 95% confidence interval). Make clear	Tables 2-5

For peer review only - http://bmjopen.bmj.com/site/about/guidelines.xhtml

		which confounders were adjusted for and why they were included	
		(*b*) Report category boundaries when continuous variables were categorized	Tables 2-5
		(*c*) If relevant, consider translating estimates of relative risk into absolute risk for a meaningful time period	N/A
Other analyses	17	Report other analyses done—eg analyses of subgroups and interactions, and sensitivity analyses	N/A
**Discussion**			
Key results	18	Summarise key results with reference to study objectives	12
Limitations	19	Discuss limitations of the study, taking into account sources of potential bias or imprecision. Discuss both direction and magnitude of any potential bias	13
Interpretation	20	Give a cautious overall interpretation of results considering objectives, limitations, multiplicity of analyses, results from similar studies, and other relevant evidence	12-13
Generalisability	21	Discuss the generalisability (external validity) of the study results	13
**Other information**			
Funding	22	Give the source of funding and the role of the funders for the present study and, if applicable, for the original study on which the present article is based	15

*Give information separately for exposed and unexposed groups.

Note: An Explanation and Elaboration article discusses each checklist item and gives methodological background and published examples of transparent reporting. The STROBE checklist is best used in conjunction with this article (freely available on the Web sites of PLoS Medicine at http://www.plosmedicine.org/, Annals of Internal Medicine at http://www.annals.org/, and Epidemiology at http://www.epidem.com/). Information on the STROBE Initiative is available at www.strobe-statement.org.

For peer review only
