# Supplementary material for: Public responses to the Salisbury Novichok incident: a cross-sectional survey of anxiety, anger, uncertainty, perceived risk and avoidance behaviour in the local community
Source: BMJ Open. 2020 Sep 25;10(9):e036071. doi: 10.1136/bmjopen-2019-036071 (PMC7520835; doi:10.1136/bmjopen-2019-036071)
Supplement: Reviewer comments [file bmjopen-2019-036071.reviewer_comments.pdf]

PEER REVIEW HISTORY

BMJ Open publishes all reviews undertaken for accepted manuscripts. Reviewers are asked to complete a checklist review form (**http://bmjopen.bmj.com/site/about/resources/checklist.pdf**) and are provided with free text boxes to elaborate on their assessment. These free text comments are reproduced below.

ARTICLE DETAILS

**TITLE (PROVISIONAL)**	Public responses to the Salisbury Novichok incident: A cross-sectional survey of anxiety, anger, uncertainty, perceived risk and avoidance behaviour in the local community
**AUTHORS**	Rubin, GJ; Webster, Rebecca; Amlot, Richard; Carter, Holly; Weston, Dale; Wessely, Simon

VERSION 1 – REVIEW

**REVIEWER**	Dr Andrew Hinde<br>University of Southampton<br>United Kingdom of Great Britain and Northern Ireland
**REVIEW RETURNED**	19-Dec-2019

**GENERAL COMMENTS**	This is a report of a cross-sectional survey of public responses to the Salisbury Novichok incident of 4 March 2018. In this incident, an assassination attempt was made on a ex-Russian intelligence officer who lived in the city of Salisbury in the United Kingdom using a nerve agent. The intelligence officer, his daughter and a police officer became seriously ill, and parts of the city centre were cordoned off with access being prohibited for some time, as contamination was feared.<br><br>The aims of the survey (and of the paper) are clearly stated. The analysis is descriptive, although logistic regression was used to adjust for age and sex when using odds ratios to reporting the impact of risk factors on outcome variables. The paper is well written and reasonably easy to read.<br><br>The results are not very surprising: females, older people and those who believed they may have been exposed to the agent were more likely to report anxiety, anger or uncertainty about the outcome. Those who believed they or others might have been exposed, and who thought the intention of the would-be assassins was to harm the wider public, were more likely to think they were at personal risk and to avoid going near the contaminated areas. Those who had heard less about the recovery effort, or who were less satisfied with what was being done, or who generally trusted the authorities less, were more likely to report anxiety, anger or uncertainty and to think they were at greater risk.<br><br>Given what happened in July 2018, people’s anxiety and uncertainty about the risk to themselves was perhaps justified. You mention the July incident on p. 12, ll. 21-7. It would have been very interesting to have repeated your survey two or three weeks after the death of Ms Sturgess to see whether the overall proportions you report in Table 1, p. 19, had changed.

	I have one major suggestion for improving the paper.<br><br>Could you say a bit more about the representativeness of your sample? You mention (p. 4, ll. 2-3 that the sample was 'demographically representative of adult residents living in Salisbury', but you provide to evidence to back this claim up. You could add a table comparing the age and sex structure of your sample with that reported for the city by the 2011 population census, at the very least. The survey was also conducted by telephone. There is a plethora of studies about the likely biases inherent in telephone surveys, much of which relates to their use in political opinion polling. I am not so much concerned about the design of the questionnaire or the actual conduct of the interviews – your surveys seems to conform pretty well to the recommendations for the actual conduct of telephone interviews. I am more concerned with the representativeness of the people you reached on matters such as economic activity (you used landlines, so people who were not an home would not answer); social activity (again, socially active people may be less likely to have landlines and, if they do have them, less likely to be at home to answer them. As you say (p. 4, ll. 13-4) 'whether the surveys are psychologically representative of the general population is unclear'. This may be so, but it is quite important for the interpretation of your results and I think you need to include more discussion of this ways in which your sample might be psychologically unrepresentative and the potential implications for the interpretation of your results.<br><br>Finally, there is some confusion over whether you use weighted or unweighted data. On p. 9, l. 26 you say that 'all analyses were conducted using unweighted data'. However on p. 25, you say that '[d]ata have been weighted' to age and sex. These two statements seem contradictory. Can you clarify this?

**REVIEWER**	Abdurrahman Altindag<br>Gaziantep University Turkey
**REVIEW RETURNED**	21-Mar-2020

**GENERAL COMMENTS**	The reviewer completed the checklist but made no further comments.

VERSION 1 – AUTHOR RESPONSE

Reviewer: 1
1. Given what happened in July 2018, people's anxiety and uncertainty about the risk to themselves was perhaps justified. You mention the July incident on p. 12, ll. 21-7. It would have been very interesting to have repeated your survey two or three weeks after the death of Ms Sturgess to see whether the overall proportions you report in Table 1, p. 19, had changed.

We agree, but regrettably did not have funding to repeat the study.

2. Could you say a bit more about the representativeness of your sample? You mention (p. 4, ll. 2-3 that the sample was 'demographically representative of adult residents living in Salisbury', but you provide to evidence to back this claim up. You could add a table comparing the age and sex structure of your sample with that reported for the city by the 2011 population census, at the very least. The survey was also conducted by telephone. There is a plethora of studies about the likely biases

inherent in telephone surveys, much of which relates to their use in political opinion polling. I am not so much concerned about the design of the questionnaire or the actual conduct of the interviews – your surveys seems to conform pretty well to the recommendations for the actual conduct of telephone interviews. I am more concerned with the representativeness of the people you reached on matters such as economic activity (you used landlines, so people who were not an home would not answer); social activity (again, socially active people may be less likely to have landlines and, if they do have them, less likely to be at home to answer them. As you say (p. 4, ll. 13-4) 'whether the surveys are psychologically representative of the general population is unclear'. This may be so, but it is quite important for the interpretation of your results and I think you need to include more discussion of this ways in which your sample might be psychologically unrepresentative and the potential implications for the interpretation of your results.

We have clarified that "the study sample was demographically representative of adult residents living in Salisbury, UK in terms of age and gender" [page four]

We have clarified in the text that "calls were made during the working day, during the evenings and at the weekend to reduce selection bias associated with working outside the home." [Methods, design section].

We have noted in first paragraph of the results section that "Two hundred and eighty participants (56.0%) were female and 220 (44.0%) male. This was roughly in line with 2011 census data for Salisbury (52% female, 48% male)[18]. One hundred and sixty-eight participants (33.6%) were aged 18 to 44, 195 (39.0%) were aged 45 to 64, and 137 (27.4%) were 65 or over. This showed an underrepresentation of 18 to 44 year olds compared to census data (43%)."

In the limitations paragraph of the discussion we have reported that "Nonetheless, the underrepresentation of people aged 18 to 44 in comparison to older age groups was notable. This may relate to a lower proportion within this group who own a landline telephone. Given that older age was associated with anxiety and anger, it could be that this bias has led us to over-estimate the psychological impact of the incident."

3. Finally, there is some confusion over whether you use weighted or unweighted data. On p. 9, l. 26 you say that 'all analyses were conducted using unweighted data'. However on p. 25, you say that '[d]ata have been weighted' to age and sex. These two statements seem contradictory. Can you clarify this?

We have clarified that "Although topline data in the supplementary materials are reported weighted, for ease of interpretation, all analyses were conducted using unweighted data."

Reviewer: 2
No comments were received

VERSION 2 – REVIEW

**REVIEWER**	Andrew Hinde<br>University of Southampton<br>United Kingdom
**REVIEW RETURNED**	03-Aug-2020

**GENERAL COMMENTS**	Thank you for addressing both of my comments: on the representativeness of the sample and on the use of weighted and unweighted data. I am content with your responses and I have no further comments.
